# Causal Inference in the Perception of Verticality

**DOI:** 10.1038/s41598-018-23838-w

**Published:** 2018-04-03

**Authors:** Ksander N. de Winkel, Mikhail Katliar, Daniel Diers, Heinrich H. Bülthoff

**Affiliations:** Department of Human Perception, Cognition, and Action, Max Planck Institute for Biological Cybernetics, Max-Planck-Ring 8, 72076 Tübingen, Germany

## Abstract

The perceptual upright is thought to be constructed by the central nervous system (CNS) as a vector sum; by combining estimates on the upright provided by the visual system and the body’s inertial sensors with prior knowledge that upright is usually above the head. Recent findings furthermore show that the weighting of the respective sensory signals is proportional to their reliability, consistent with a Bayesian interpretation of a vector sum (Forced Fusion, FF). However, violations of FF have also been reported, suggesting that the CNS may rely on a single sensory system (Cue Capture, CC), or choose to process sensory signals based on inferred signal causality (Causal Inference, CI). We developed a novel alternative-reality system to manipulate visual and physical tilt independently. We tasked participants (n = 36) to indicate the perceived upright for various (in-)congruent combinations of visual-inertial stimuli, and compared models based on their agreement with the data. The results favor the CI model over FF, although this effect became unambiguous only for large discrepancies (±60°). We conclude that the notion of a vector sum does not provide a comprehensive explanation of the perception of the upright, and that CI offers a better alternative.

## Introduction

Whenever we specify objects’ relative locations using terms as ‘above’ or ‘below’, or when we move throughout the world while trying not to topple over, we make use of the fact that we have a perception of upright. Multiple sensory systems throughout the body provide the nervous system with information that can potentially be used to construct a subjective vertical: visually, we are able to determine our orientation from polarity information in the optic array^1^; our vestibular system is stimulated by accelerations, and therefore provides us with information on the direction and magnitude of the gravitational vector^2^; we receive information about our orientation relative to gravity from pressure cues on the body^3,4^ and the distribution of fluids in the body^5,6^; and there is evidence for specialized graviceptors located in the trunk^7^.

Mittelstaedt^8^ proposed that the Central Nervous System (CNS) constructs perceptions of verticality by combining the sensory information from the visual system and the body’s collective inertial sensors with the prior knowledge that ‘up’ is usually aligned with the long-body axis (the idiotropic vector), and that the process could be described as a vector sum, where the length of the vectors represents the relative influence of each component. In subsequent work, this concept has been interpreted as a reflection of statistically optimal behavior by the Central Nervous System (CNS): according to Bayes’ rule, if sensory estimates of the upright are normally distributed random variables and the prior is either normally distributed or uninformative, the estimate that is most likely the true upright can be calculated as a weighted average of the sensory estimates, where the weights are proportional to the inverse of estimates’ variances^9–13^.

Several studies report that people’s perception of the upright indeed reflects such Bayesian integration^14–18^. Different measures were used among studies: participants were instructed to either indicate the Subjective Visual Vertical (SVV) by aligning an object in the visual display with the perceived upright^14,16–18^; the perceptual upright was inferred from participants’ interpretations of the ambiguous symbol ‘p’, which is defined by its orientation relative to the perceived upright (equivalently, ‘d’; Oriented CHAracter Recognition Test, or OCHART^14,19^); and estimates of upright have been derived from a discrimination task, where participants discriminated between roll stimuli on the basis of Subjective Body Tilt^17^ (SBT). Although biases and intersensory weightings appear to differ between tasks^20^, the ratios of the weights attributed to the constituent cues coincided well with calculations of sensory variance for the former measures, thereby providing supporting evidence for the Bayesian interpretation of the vector-sum model; and for the latter measure, supporting evidence was obtained by comparisons of model fit indices.

However, some findings are inconsistent with such modeling. First, the weightings reported differ between experiments and appear to depend on the specific measure^14,18^. From the perspective of a Bayesian vector sum model, this means that the sensory variances differ between tasks and experiments. Even though it is not unlikely that specific conditions of an experiment affect the sensory estimates, it is not clear why the variance of sensory estimates of the upright would vary depending on the task. Second, De Winkel *et al*.^21^ performed a study where participants were asked to report the SVV during exposure to different levels of gravity during parabolic flight. Here, it was found that participants discarded the visual cue entirely, and relied on either inertial or idiotropic information in a dichotomous fashion, where the probability of relying on the inertial cue was proportional to the strength of the gravitational pull.

The observed differences in the role specific cues fulfill in different tasks and the aforementioned variability in sensory weightings imply that Bayesian models based on the notion of a vector sum cannot offer a comprehensive explanation of the perception of the upright. Consistent with recent reports on audiovisual interactions in spatial localization tasks^22–25^ and visual-inertial heading estimation^26,27^, it is possible that the role attributed to different cues reflects the inferred causality of the signals. Models that can account for different behaviors depending on inferred causality are Causal Inference (CI) models^22,23^. Put simply, these models state that the CNS constructs intermediate estimates of environmental properties consistent with different interpretations of their causes (i.e., a common cause or separate causes) in tandem, and combines these into final estimates, taking into account a-priori beliefs on the probability of alternative causal structures.

We hypothesized that CI models provide a better explanation of the perceptual upright than the vector-sum approach. In an experiment, we independently manipulated participants’ physical and visual orientation with respect to the true vertical, and tasked them to provide estimates of what they thought was the true, physical, upright. We developed statistical versions of prominent models of multisensory perception and compared their ability to explain the participants’ responses.

## Methods

### Overview

To test the hypothesis outlined above experimentally, we placed 36 participants on a motion platform that was capable of physically rotating them around their naso-occipital axis. While seated on this platform, participants wore a head-mounted display (HMD) setup that allowed us to manipulate visual orientation independently from the true gravitational vertical. Participants indicated what they thought was upright for various (in)congruent visual-inertial orientation stimuli. We assessed how well a number of alternative models of spatial orientation could account for participants’ responses by fitting each model to the data and comparing model fit indices.

### Ethics statement

The experiment was carried out in accordance with the declaration of Helsinki. All participants provided written informed consent prior to participation. The experimental protocol was approved by the ethical committee of the medical faculty of the Eberhard-Karls University in Tübingen, Germany, reference number 352/2017BO2.

### Data availability statement

All experimental data are available as supplementary material Data S1.

### Participants

A total of 40 participants were recruited for the experiment. Two participants were not able to complete the experiment due to motion sickness; data from two other participants had to be excluded due to an inability to perform the task. Of the remaining 36 participants, 20 were male. The mean age was 29.5 years, with a standard deviation of 7.3. Participants 11, 14, and 16 in the first iteration of the experiment also participated as participants 34, 36, and 30 in the third iteration. Participants were compensated for their time at a rate of €8 an hour.

### Setup

**Figure 1:**
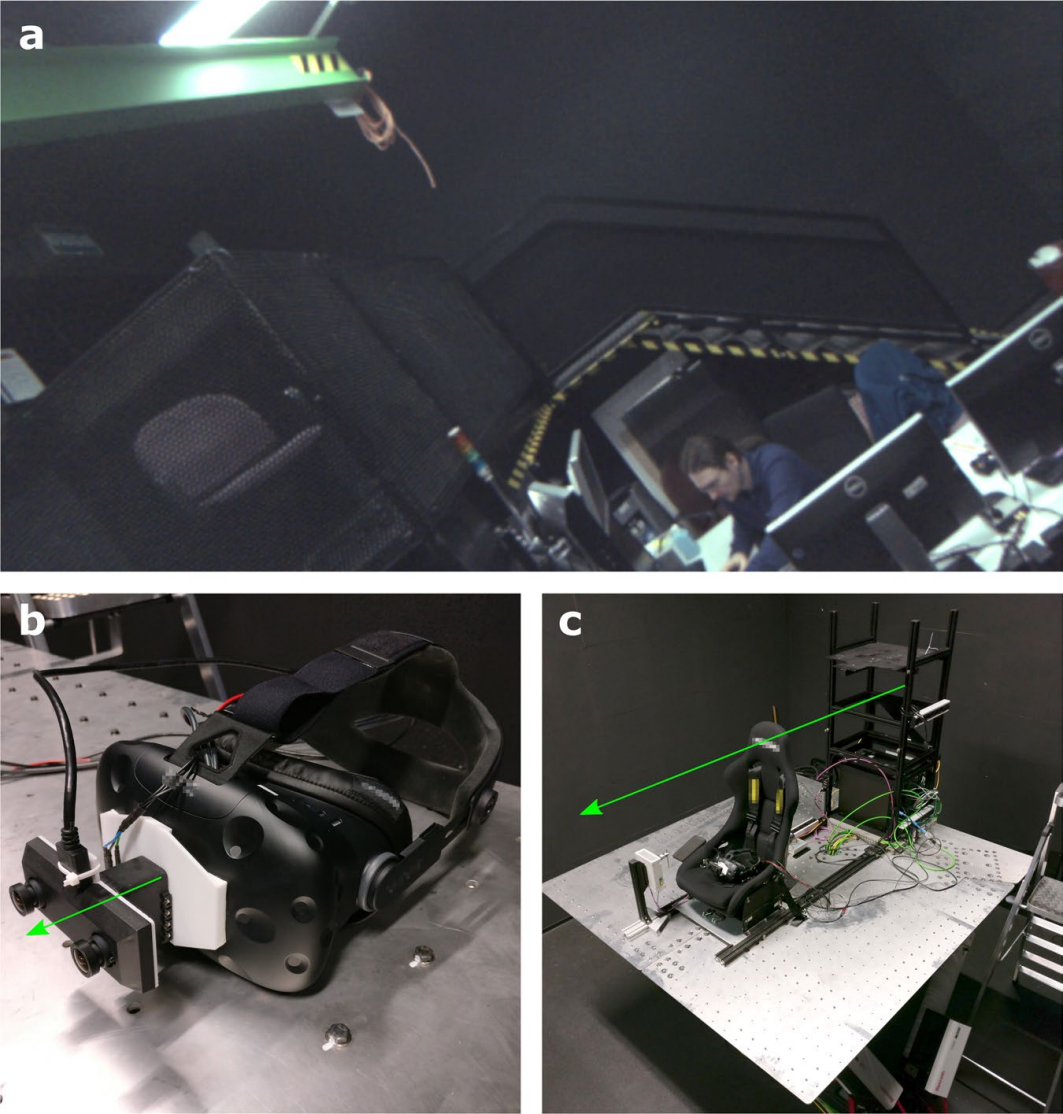
(**a**) (Monoscopic) screenshot of a participant’s view through the alternative-reality system, showing the entrance and control area of the simulator hall. (**b**) photograph of the alternative-reality system. (**c**) view of the experimental setup, showing the motion platform and seat. The green arrows indicate axes of rotation. The pointer device is on the right hand side of the seat.

Visual stimuli were generated and presented using a custom-made alternative-reality setup. This setup allowed us to show participants their immediate visual environment at any desired roll-tilt angle stereoscopically and in real-time, such that the visual stimulus was equivalent to visual tilt experienced due to head tilt. The system was designed with the aim to maximize the ecological validity of the visual stimuli as a cue to orientation; to avoid the possibility that they would be discarded simply because they are unlikely to reflect one’s spatial orientation^21^. For instance, when reading a large book while lying on one’s side, the orientation of the book does not reflect one’s own orientation. Through this system, participants viewed the entrance and control area of the simulator hall. The visible area of the room was approximately 5 m wide by 4 m high and 5 m deep, and included a green crane, stairs, the platform control systems, and the experimenter. The elevated platform is 2 m high, and the nearest edge was 2 m in front of the participant. The visual stimulus could be used to estimate the upright by means of polarity cues, support relationships between objects, and motion of the experimenter (e.g.^2,28^). A (monoscopic) screenshot of the participants’ view is presented in the top panel of Fig. 1.

The alternative-reality system system consisted of an OVRVision Pro stereo camera (Wizapply, Osaka, Japan) mounted via a Dynamixel AX12-A servo motor (Robotis, Lake Forest, California, United States) to a Vive HMD (HTC, New Taipei City, Taiwan). Mounting hardware was designed and 3D-printed in-house. The HMD displayed the images of the left and right camera in the respective screens at a rate of 45 frames per second. The screens each have a resolution of 1080 × 1200 px and a field of view of 100 × 110°, corresponding to approximately 11 px per degree. The servo allowed us to manipulate the orientation of the camera with an accuracy of 0.29°. Note that, being equivalent to head-tilt, a clockwise tilt of the camera results in the perception of a counter-clockwise rotation of the visual environment. The software to control the servo and transmit the camera images was also developed in-house. The device is shown in Fig. 1, bottom left panel.

Inertial orientation stimuli were presented using an eMotion 1500 hexapod motion system (Bosch Rexroth AG, Lohr am Main, Germany). For different physical orientation stimuli, the platform was moved in such a way that the axis of rotation coincided with each participant’s naso-occipital axis. This limited the range of possible physical tilt angles to approximately ±13°. The platform was controlled using Simulink software (The MathWorks, Inc., Natick, Massachusetts, United States). Participants were seated in an automotive style bucket seat (RECARO GmbH, Stuttgart, Germany) that was mounted on top of the platform, and secured with a 5-point safety harness (SCHROTH Safety Products GmbH, Arnsberg, Germany). To minimize head movements, participants also wore a Philadelphia type cervical collar. To ensure that participants could not simply see any tilt of the platform relative to the room, we moved the chair to the edge of the platform. To mask the sounds of the motion platform and the servo-motor, participants wore earplugs with a 33 dB signal-to-noise ratio (Honeywell Safety Products, Roissy, France) as well as a wireless headset (Plantronics, Santa Cruz, California, United States) that actively canceled outside noise, and that played white noise during platform rotations. A photograph of the complete setup is presented in the bottom right panel of Fig. 1.

### Task and Stimuli

In each trial, we manipulated the orientation of the visual environment using the alternative-reality system, and the participants’ physical orientation by tilting the motion platform. Participants were tasked to align a pointer device with the perceived physical ‘up’ (the negative of the perceived tilt) on a large number of experimental trials. This method is known as setting the Subjective Haptic Vertical (SHV).

The pointer device consisted of a 15 cm stainless steel rod mounted to a potentiometer. About $\tfrac{1}{5}$ of the length of the rod extended above its center of rotation. The short hand was to be interpreted as the pointer’s top-end, and had to be pointed upwards. The pointer device was free of discontinuities, was not affected by rotation relative to gravity, and provided a <0.1° resolution. Participants registered a pointer setting as a response by pressing a button at the base of the rod.

There were 25 experimental conditions, comprising all possible combinations of five visual (*θ*_*V*_) and five physical (*θ*_*I*_) roll-tilt angles. The specifics of the stimuli were adjusted in three subsequent iterations of the experiment. These adjustments were made to improve discriminability of the statistical models. In the first iteration of the experiment (participants 1–19), *θ*_*V*_ and *θ*_*I*_ both had values of [−10, −5, 0, 5, 10]°, where 0° corresponds to the gravitational vertical (maximum discrepancy ±20°); in the second iteration (participants 20–28), the range of angles was slightly inflated, to [−13, −6.5, 0, 6.5, 13]° (maximum discrepancy ±26°); and in the final iteration (participants 29–36), *θ*_*V*_ had values of [−50, −25, 0, 25, 50]°, and *θ*_*I*_ had values of [−10, −5, 0, 5, 10]° (maximum discrepancy ±60°). In the first and second iteration of the experiment, there were 16 repetitions of each condition, totaling 400 trials; in the third iteration there were 21 repetitions per condition, totaling 525 trials. Note that: *θ*_*V*_ is equal to the sum of the physical tilt and tilt of the camera setup (*θ*_*I*_ = *θ*_*I*_ + *θ*_HMD_); equal values for the visual and physical roll tilt angle indicate that the camera was aligned with the participants’ physical orientation relative to gravity (i.e., the visual and inertial cue were congruent); and positive angles correspond to clockwise rotations.

To ensure the independence of trials, we tested three methods of transitioning from the visual and physical orientations on one trial to the next during the first iteration of the experiment. For the first nine participants, the camera was turned off while the platform was moved. For the latter five of these participants, heave and sway vibrations were added to the motion profile. These vibrations were in the range of 4–8 Hz and had a root mean square of approximately 0.1 m/s^2^. These vibrations are comparable to road rumble. For the remainder of the participants, the camera was always on during transitions, and there were no vibrations. Regardless of the transition method, the velocity profile of the roll-rotation followed a raised cosine bell, with a duration that was randomly varied between 3–4 s. We did not find any differences in the results of these different subgroups.

Including instructions and 5-minute breaks every 15 minutes, the experiment lasted between two and three hours.

### Models of Spatial Orientation

The aim of the present modeling efforts is to assess the tenability of a number of prominent theories on how the Central Nervous System (CNS) constructs perceptions of upright under conditions of uncertainty about the causality of potential cues on the upright.

We postulate that a response *R* reflecting the perceived upright is equal to the negative of the final tilt estimate *r* (*R* = −*r*), and that *r* is constructed from visual (V) and inertial (I) sensory estimates of orientation (*x*_*V*_, *x*_*I*_). The visual system can generate estimates of orientation using polarity information that is present in the optical array (e.g., blue sky/green grass; objects lying on a shelf). Our body’s inertial sensors comprise the vestibular system of the inner ear and various kinds of other sensory neurons distributed throughout the body. Because all these neurons are, either directly or indirectly, responsive to accelerations, we treat them as one single inertial system. The inertial sensory system can generate estimates of orientation by identifying the direction of gravity.

For either sensory modality *m* ∈ {V, I}, we assume orientation estimates *x*_*m*_, are realizations of a random variable that is a possibly distorted version of the respective actual tilt *θ*_*m*_. We further assume that for the presently investigated range of orientations around the true gravitational upright, the noise can be approximated by a Gaussian distribution with standard deviation *σ*_*m*_, and that the distortion can be expressed with a scaling parameter *β*_*m*_.

1 $${\rm{P}}({x}_{m}|{\theta }_{m})=\frac{1}{\sqrt{2\pi }{\sigma }_{m}}\,\exp \,(-\frac{{({x}_{m}-{\beta }_{m}{\theta }_{i})}^{2}}{2{\sigma }_{m}^{2}}).$$

Note that roll-tilt is a circular variable, which should ideally be represented by circular distributions^26,29,30^. Predictions on perception from cue combination (fusion) models using circular distributions diverge from those of models using Normal distributions when realizations of variables cross the extremes of the circle (±180°), and as a function of intersensory discrepancy. More specifically, whereas sensory weightings and the standard deviation of the integrated percept are unaffected by discrepancies in models based on Normal distributions, discrepancies bias the integrated percept towards the more certain sensory estimate in a model using Von Mises distributions, and the uncertainty of the integrated estimate increases as a function of the size of intersensory discrepancy. For a detailed account on cue combination for circular variables and differences between model predictions see Murray and Morgenstern^29^. We evaluated differences between predictions of a fusion model using Normal and Von Mises distributions using the average values for the standard deviations of the visual and inertial estimates (as per Table 2) and the maximum discrepancy from the corresponding iteration. The overlap between response densities according to the two different models, expressed as the Bhattacharyya distance^31^, was consistently above 0.99, where 1 is perfect overlap, and 0 is no overlap at all. The predictions on means differed by 0.036°, 0.039°, and 0.498°; and the standard deviations by 0.027°, 0.072°, and 0.820°, for the three iterations, respectively. Because the differences were negligible, we used Gaussian distributions. This allowed us to formulate analytical expressions for the models and to fit them to the data without the need to resort to simulations and numerical integration.

We corrected for constant offset in the responses by calibrating the rod zero-position with the subjective upright at the outset of the experiment, and by subtracting the mean from the data.

We do not have access to the sensory estimates *x*_*m*_, and are interested in the probability of the tilt estimate *r* given stimuli *θ*_*V*_, *θ*_*I*_. This probability is

2 $$\begin{array}{rcl}{\rm{P}}(r|{\theta }_{V},{\theta }_{I}) & = & {\iint }_{-\infty }\,{\rm{P}}(r,{x}_{V},{x}_{I}|{\theta }_{V},{\theta }_{I})\,{\rm{d}}{x}_{V}\,{\rm{d}}{x}_{I}\\  & = & {\iint }_{-\infty }\,{\rm{P}}(r|{x}_{V},{x}_{I},{\theta }_{V},{\theta }_{I})\,{\rm{P}}({x}_{V},{x}_{I}|{\theta }_{V},{\theta }_{I})\,{\rm{d}}{x}_{V}\,{\rm{d}}{x}_{I}.\end{array}$$

We assume that the sensory estimates for different modalities are generated independently. Therefore, ${\rm{P}}({x}_{V},{x}_{I}|{\theta }_{V},{\theta }_{I})={\rm{P}}({x}_{V}|{\theta }_{V})\,{\rm{P}}({x}_{I}|{\theta }_{I})$ and the equation above can be rewritten as

3 $${\rm{P}}(r|{\theta }_{V},{\theta }_{I})={\iint }_{-\infty }\,{\rm{P}}(r|{x}_{V},{x}_{I},{\theta }_{V},{\theta }_{I})\,{\rm{P}}({x}_{V}|{\theta }_{V})\,{\rm{P}}({x}_{I}|{\theta }_{I})\,{\rm{d}}{x}_{V}\,{\rm{d}}{x}_{I}.$$

Since *x*_*V*_ and *x*_*I*_ is the only information available to the observer, *r* will be conditionally independent of other variables apart from *x*_*V*_ and *x*_*I*_, i.e.,

4 $${\rm{P}}(r|{x}_{V},{x}_{I},{\theta }_{V},{\theta }_{I})={\rm{P}}(r|{x}_{V},{x}_{I}\mathrm{)}.$$

By taking this into account and substituting (1) into (3), we get

5 $$\begin{array}{rcl}{\rm{P}}(r|{\theta }_{V},{\theta }_{I}) & = & \frac{1}{2\pi {\sigma }_{I}{\sigma }_{V}}\,{\iint }_{-\infty }\,{\rm{P}}(r|{x}_{V},{x}_{I})\\  &  & \times \exp \,(-\frac{{({x}_{I}-{\beta }_{I}{\theta }_{I})}^{2}}{2{\sigma }_{I}^{2}}-\frac{{({x}_{V}-{\beta }_{V}{\theta }_{V})}^{2}}{2{\sigma }_{V}^{2}})\,{\rm{d}}{x}_{V}\,{\rm{d}}{x}_{I}.\end{array}$$

Because a participant is unaware of *θ*_*m*_ and *β*_*m*_, the final tilt estimate generation model P(*r*|*x*_*m*_) uses a different sensory estimate (*x*_*m*_) generation model than the true sensory estimate generation model.

From a participant’s perspective, the likelihood of the sensory estimate given any orientation ${\rm{\Theta }}$ being the true orientation can be expressed as

6 $${\rm{P}}({x}_{m}|{\rm{\Theta }})=\frac{1}{\sqrt{2\pi }{\sigma }_{m}}\,\exp \,(-\frac{{({x}_{m}-{\rm{\Theta }})}^{2}}{2{\sigma }_{m}^{2}}).$$

Moreover, we consider the notion that the CNS includes a-priori beliefs about ${\rm{\Theta }}$, namely that we are usually upright, in the construction of this percept. We choose the long body axis as the reference (0°) for other angles. Consequently, we define a prior belief of the following form:

7 $${\rm{P}}({\rm{\Theta }})=\frac{1}{\sqrt{2\pi }{\sigma }_{0}}\,\exp \,(-\frac{{{\rm{\Theta }}}^{2}}{2{\sigma }_{0}^{2}}),$$

where *σ*_0_ is the distribution’s standard deviation, which represents the strength of the prior belief. In accordance with the literature, we refer to this prior as the ‘idiotropic prior’.

Various general strategies have been proposed on how the CNS may construct final tilt estimates from the multisensory estimates and prior beliefs. Below, we provide mathematical formulations of prominent strategies.

#### Cue Capture

According to Cue Capture (CC) models, perception of specific environmental properties is dominated by a single sensory modality^32–34^. In the present case, there are two such possibilities: perception of the upright is dominated by either visual or inertial information. A prior belief that the upright aligns with the long body axis interacts with the sensory information according to Bayes’ rule. The posterior probability of Θ given either sensory estimate *x*_*m*_ is then given by:

8 $$\begin{array}{rcl}{\rm{P}}({\rm{\Theta }}|{x}_{m}) & = & \frac{{\rm{P}}({x}_{m}|{\rm{\Theta }})\,{\rm{P}}({\rm{\Theta }})}{{\rm{P}}({x}_{m})}\propto \exp \,(-\frac{{({x}_{m}-{\rm{\Theta }})}^{2}}{2{\sigma }_{m}^{2}})\,\exp \,(-\frac{{{\rm{\Theta }}}^{2}}{2{\sigma }_{0}^{2}})\\  & = & \exp \,(-\frac{{({x}_{m}-{\rm{\Theta }})}^{2}}{2{\sigma }_{m}^{2}}-\frac{{{\rm{\Theta }}}^{2}}{2{\sigma }_{0}^{2}}).\end{array}$$

Consistent with the literature, we assume that for individual trials *r* is the mode of this posterior distribution (i.e., the Maximum-A-Posteriori estimate, MAP)

9 $${r}_{{\rm{CC}}}({x}_{m})=\mathop{{\rm{argmax}}}\limits_{{\rm{\Theta }}}\,{\rm{P}}({\rm{\Theta }}|{x}_{m})=\frac{{\sigma }_{0}^{2}{x}_{m}}{{\sigma }_{0}^{2}+{\sigma }_{m}^{2}}={\alpha }_{m}{x}_{m}$$

where ${\alpha }_{m}=\tfrac{{\sigma }_{0}^{2}}{{\sigma }_{0}^{2}+{\sigma }_{m}^{2}}$. The corresponding PDF can be expressed as

10 $${\rm{P}}({r}_{{\rm{CC}}}|{x}_{m})=\delta ({r}_{{\rm{CC}}}-{\alpha }_{m}{x}_{m})$$

in which *δ*(·) is Dirac’s delta function. By substituting (10) into (5) we obtain

11 $$\begin{array}{rcl}{\rm{P}}({r}_{{\rm{CC}}}|{\theta }_{m}) & = & \frac{1}{\sqrt{2\pi }{\sigma }_{m}}\,{\int }_{-\infty }^{\infty }\,\delta ({r}_{{\rm{CC}}}-{\alpha }_{m}{x}_{m})\,\exp \,(-\frac{{({x}_{m}-{\beta }_{m}{\theta }_{m})}^{2}}{2{\sigma }_{m}^{2}})\,{\rm{d}}{x}_{m}\\  & = & \frac{1}{\sqrt{2\pi }{\alpha }_{m}{\sigma }_{m}}\,\exp \,(-\frac{{({r}_{{\rm{CC}}}-{\alpha }_{m}{\beta }_{m}{\theta }_{m})}^{2}}{2{\alpha }_{m}^{2}{\sigma }_{m}^{2}}).\end{array}$$

#### Switching Strategy

The CC models can be considered special cases of the Switching Strategy (SS) model^21,34^. The SS model essentially combines the two alternative CC models: the CNS constructs *r* for each sensory modality as in the Cue Capture models, but randomly chooses either modality as dominant source on a trial-by-trial basis:

12 $${r}_{{\rm{SS}}}({x}_{V},{x}_{I})=(\begin{array}{ll}{\alpha }_{V}{x}_{V}, & {\rm{with}}\,{\rm{probability}}\,{\rm{P}}({\rm{V}})\\ {\alpha }_{I}{x}_{I}, & {\rm{with}}\,{\rm{probability}}\,1-{\rm{P}}({\rm{V}})\end{array}$$

with *α*_*m*_ as before, with the corresponding probability density function

13 $${\rm{P}}({r}_{{\rm{SS}}}|{x}_{I},{x}_{V})=P(V)\delta ({r}_{{\rm{SS}}}-{\alpha }_{V}{x}_{V})+\mathrm{(1}-P(V))\delta ({r}_{{\rm{SS}}}-{\alpha }_{I}{x}_{I}\mathrm{)}.$$

Filling in (13) into (5), we obtain the likelihood of the responses given the stimuli P(*r*_SS_|*θ*_*V*_,*θ*_*I*_):

14 $$\begin{array}{rcl}{\rm{P}}({r}_{{\rm{SS}}}|{\theta }_{V},{\theta }_{I}) & = & \frac{{\rm{P}}({\rm{V}})}{\sqrt{2\pi }{\alpha }_{V}{\sigma }_{V}}\,\exp \,(-\frac{{({r}_{{\rm{SS}}}-{\alpha }_{V}{\beta }_{V}{\theta }_{V})}^{2}}{2{\alpha }_{V}^{2}{\sigma }_{V}^{2}})\\  &  & +\frac{1-{\rm{P}}({\rm{V}})}{\sqrt{2\pi }{\alpha }_{I}{\sigma }_{I}}\,\exp \,(-\frac{{({r}_{{\rm{SS}}}-{\alpha }_{I}{\beta }_{I}{\theta }_{I})}^{2}}{2{\alpha }_{I}^{2}{\sigma }_{I}^{2}}).\end{array}$$

#### Forced Fusion

In the present formulation of the Forced Fusion (FF) model, it is assumed that visual and inertial sensory estimates are independent from each other, and that both are always interpreted as cues to orientation^9–13^. The posterior probability of Θ can be expressed as:

15 $$\begin{array}{rcl}{\rm{P}}({\rm{\Theta }}|{x}_{V},{x}_{I}) & = & \frac{{\rm{P}}({x}_{V},{x}_{I}|{\rm{\Theta }})\,{\rm{P}}({\rm{\Theta }})}{{\rm{P}}({x}_{V},{x}_{I})}\\  & \propto  & \exp \,(-\frac{{({x}_{V}-{\rm{\Theta }})}^{2}}{2{\sigma }_{V}^{2}})\,\exp \,(-\frac{{({x}_{I}-{\rm{\Theta }})}^{2}}{2{\sigma }_{I}^{2}})\,\exp \,(-\frac{{{\rm{\Theta }}}^{2}}{2{\sigma }_{0}^{2}})\\  & = & \exp \,(-\frac{{({x}_{V}-{\rm{\Theta }})}^{2}}{2{\sigma }_{V}^{2}}-\frac{{({x}_{I}-{\rm{\Theta }})}^{2}}{2{\sigma }_{I}^{2}}-\frac{{{\rm{\Theta }}}^{2}}{2{\sigma }_{0}^{2}}).\end{array}$$

The final tilt estimate *r*_FF_ is again the mode of the posterior distribution (the MAP):

16 $${r}_{{\rm{FF}}}({x}_{V},{x}_{I})=\mathop{{\rm{argmax}}}\limits_{{\rm{\Theta }}}\,{\rm{P}}{\rm{r}}({\rm{\Theta }}|{x}_{V},{x}_{I})={\alpha }_{0V}\,{x}_{V}+{\alpha }_{0I}\,{x}_{I},$$

with ${\alpha }_{0V}=\frac{{\sigma }_{0}^{2}{\sigma }_{I}^{2}}{{\sigma }_{0}^{2}{\sigma }_{I}^{2}+{\sigma }_{0}^{2}{\sigma }_{V}^{2}+{\sigma }_{I}^{2}{\sigma }_{V}^{2}}$, and ${\alpha }_{0I}=\frac{{\sigma }_{0}^{2}{\sigma }_{V}^{2}}{{\sigma }_{0}^{2}{\sigma }_{I}^{2}+{\sigma }_{0}^{2}{\sigma }_{V}^{2}+{\sigma }_{I}^{2}{\sigma }_{V}^{2}}$. Similar to (10), the posterior PDF can be expressed as

17 $${\rm{P}}({r}_{{\rm{FF}}}|{x}_{V},{x}_{I})=\delta ({r}_{{\rm{FF}}}-{\alpha }_{0V}\,{x}_{V}-{\alpha }_{0I}\,{x}_{I}).$$

By substituting (17) into (5) and subsequently simplifying, we obtain

18 $$\begin{array}{rcl}{\rm{P}}({r}_{{\rm{FF}}}|{\theta }_{V},{\theta }_{I}) & = & \frac{1}{2\pi {\sigma }_{V}{\sigma }_{I}}\,{\int }_{-\infty }^{\infty }\,{\int }_{-\infty }^{\infty }\,\delta ({r}_{{\rm{FF}}}-{\alpha }_{0V}{x}_{V}-{\alpha }_{0I}{x}_{I})\\  &  & \exp \,(-\frac{{({x}_{V}-{\beta }_{V}{\theta }_{V})}^{2}}{2{\sigma }_{V}^{2}}-\frac{{({x}_{I}-{\beta }_{I}{\theta }_{I})}^{2}}{2{\sigma }_{I}^{2}})\,{\rm{d}}{x}_{V}\,{\rm{d}}{x}_{I}\\  & = & \frac{1}{\sqrt{2\pi }{\sigma }_{{\rm{FF}}}}\,\exp \,(-\frac{{({r}_{{\rm{FF}}}-{\mu }_{{\rm{FF}}})}^{2}}{2{\sigma }_{{\rm{FF}}}^{2}}),\end{array}$$

where

19 $${\mu }_{{\rm{FF}}}={\beta }_{I}{\theta }_{I}{\alpha }_{0I}+{\beta }_{V}{\theta }_{V}{\alpha }_{0V},$$

20 $${\sigma }_{{\rm{FF}}}^{2}={\sigma }_{I}^{2}{\alpha }_{0I}^{2}+{\sigma }_{V}^{2}{\alpha }_{0V}^{2}.$$

#### Causal Inference Model

In the models presented above, it is assumed that the CNS either segregates (CC, SS) or fuses multisensory information (FF). CI models allow segregation and fusion to occur in tandem; the estimates generated by the different strategies are treated as intermediate estimates, and a final estimate is constructed by taking into account the probability that the internal estimates share a common cause (C), favoring FF; or have independent causes ($\overline{{\rm{C}}}$), favoring SS^22,23,25,26,30^.

The probability of a common cause given the sensory estimates is

21 $$\begin{array}{rcl}{\rm{P}}({\rm{C}}|{x}_{V},{x}_{I}) & = & \frac{{\rm{P}}({x}_{V},{x}_{I}|{\rm{C}})\,{\rm{P}}({\rm{C}})}{{\rm{P}}({x}_{V},{x}_{I})}\\  & = & \frac{{\rm{P}}({x}_{V},{x}_{I}|{\rm{C}})\,{\rm{P}}({\rm{C}})}{{\rm{P}}({x}_{V},{x}_{I}|{\rm{C}})\,{\rm{P}}({\rm{C}})+{\rm{P}}({x}_{V},{x}_{I}|\bar{{\rm{C}}})\,\mathrm{(1}-{\rm{P}}({\rm{C}}))}\end{array}$$

where P(C) is a free parameter that represents a prior tolerance for discrepancies. The likelihood of the sensory estimates *x*_*V*_, *x*_*I*_ given a common cause C is

22 $${\rm{P}}({x}_{V},{x}_{I}|{\rm{C}})={\int }_{-\infty }^{\infty }\,{\rm{P}}({x}_{V},{x}_{I}|{\rm{\Theta }})\,{\rm{P}}({\rm{\Theta }}){\rm{\Theta }}.$$

where ${\rm{P}}({\rm{\Theta }})$ is the idiotropic prior. ${\rm{P}}({x}_{V},{x}_{I}|{\rm{\Theta }})$ is the likelihood of *x*_*v*_, *x*_*I*_ given some common orientation ${\rm{\Theta }}$. This becomes

23 $$\begin{array}{ccc}{\rm{P}}({x}_{V},{x}_{I}|{\rm{C}}) & = & \frac{1}{{(\sqrt{2\pi })}^{3}{\sigma }_{V}{\sigma }_{I}{\sigma }_{0}}\,{\int }_{-{\rm{\infty }}}^{{\rm{\infty }}}\,\exp \,(-\frac{{({x}_{V}-{\rm{\Theta }})}^{2}}{2{\sigma }_{V}^{2}}-\frac{{({x}_{I}-{\rm{\Theta }})}^{2}}{2{\sigma }_{I}^{2}}-\frac{{{\rm{\Theta }}}^{2}}{2{\sigma }_{0}^{2}})\,{\rm{d}}{\rm{\Theta }}\\  & = & \frac{1}{2\pi \sqrt{{\sigma }_{V}^{2}{\sigma }_{I}^{2}+{\sigma }_{V}^{2}{\sigma }_{0}^{2}+{\sigma }_{I}^{2}{\sigma }_{0}^{2}}}\,\exp \,(-\frac{1}{2}\frac{{({x}_{V}-{x}_{I})}^{2}{\sigma }_{0}^{2}+{x}_{V}^{2}{\sigma }_{I}^{2}+{x}_{I}^{2}{\sigma }_{V}^{2}}{{\sigma }_{V}^{2}{\sigma }_{I}^{2}+{\sigma }_{V}^{2}{\sigma }_{0}^{2}+{\sigma }_{I}^{2}{\sigma }_{0}^{2}}).\end{array}$$

For an interpretation of independent causes, *r* will be based on either the visual or the inertial estimate. We assume that the same idiotropic prior interacts with both sensory estimates, as only one of them will ultimately be treated as informative of body tilt.

24 $$\begin{array}{ccc}{\rm{P}}({x}_{V},{x}_{I}|\bar{{\rm{C}}}) & = & \frac{1}{2\pi {\sigma }_{V}{\sigma }_{0}}\,{\int }_{-{\rm{\infty }}}^{{\rm{\infty }}}\,\exp \,(-\frac{{({x}_{V}-{\rm{\Theta }})}^{2}}{2{\sigma }_{V}^{2}}-\frac{{{\rm{\Theta }}}^{2}}{2{\sigma }_{0}^{2}})\,{\rm{d}}{\rm{\Theta }}\\  &  & \times \frac{1}{2\pi {\sigma }_{I}{\sigma }_{0}}\,{\int }_{-{\rm{\infty }}}^{{\rm{\infty }}}\,\exp \,(-\frac{{({x}_{I}-{\rm{\Theta }})}^{2}}{2{\sigma }_{I}^{2}}-\frac{{{\rm{\Theta }}}^{2}}{2{\sigma }_{0}^{2}})\,{\rm{d}}{\rm{\Theta }}\\  & = & \frac{1}{2\pi \sqrt{({\sigma }_{V}^{2}+{\sigma }_{0}^{2})\,({\sigma }_{I}^{2}+{\sigma }_{0}^{2})}}\,\exp \,(-\frac{1}{2}(\frac{{x}_{V}^{2}}{{\sigma }_{V}^{2}+{\sigma }_{0}^{2}}+\frac{{x}_{I}^{2}}{{\sigma }_{I}^{2}+{\sigma }_{0}^{2}})).\end{array}$$

As in^22,24^, *r*_CI_ is a weighted average of the intermediate tilt estimates according to the SS strategy *r*_SS_ and according to the FF strategy *r*_FF_, with weights proportional to the respective probability of the causal structures

25 $${r}_{{\rm{CI}}}({x}_{V},{x}_{I})={\rm{P}}(\bar{{\rm{C}}}|{x}_{V},{x}_{I})\,{r}_{{\rm{SS}}}({x}_{V},{x}_{I})+{\rm{P}}({\rm{C}}|{x}_{V},{x}_{I})\,{r}_{{\rm{FF}}}({x}_{V},{x}_{I})$$

where ${\rm{P}}(\overline{{\rm{C}}}|{x}_{V},{x}_{I}))=1-{\rm{P}}({\rm{C}}|{x}_{V},{x}_{I})$. *r*_FF_ is a deterministic function of (*x*_*V*_, *x*_*I*_) and *r*_SS_ is a random variable which can take one of the two values according to (12). Therefore, *r*_CI_ can be written as

26 $$\begin{array}{ccc}{r}_{{\rm{C}}{\rm{I}}}({x}_{V},{x}_{I}) & = & {\rm{P}}({\rm{C}}|{x}_{V},{x}_{I})\,{r}_{{\rm{F}}{\rm{F}}}({x}_{V},{x}_{I})\\  &  & +(1-{\rm{P}}({\rm{C}}|{x}_{V},{x}_{I}))(\begin{array}{cc}{\alpha }_{V}{x}_{V}, & {\rm{w}}{\rm{i}}{\rm{t}}{\rm{h}}\,{\rm{p}}{\rm{r}}{\rm{o}}{\rm{b}}{\rm{a}}{\rm{b}}{\rm{i}}{\rm{l}}{\rm{i}}{\rm{t}}{\rm{y}}\,{\rm{P}}({\rm{V}})\\ {\alpha }_{I}{x}_{I}, & {\rm{w}}{\rm{i}}{\rm{t}}{\rm{h}}\,{\rm{p}}{\rm{r}}{\rm{o}}{\rm{b}}{\rm{a}}{\rm{b}}{\rm{i}}{\rm{l}}{\rm{i}}{\rm{t}}{\rm{y}}\,1-{\rm{P}}({\rm{V}})\end{array}\end{array}$$

which can be expressed as the density function

27 $${\rm{P}}({r}_{{\rm{CI}}}|{x}_{V},{x}_{I})={\rm{P}}({\rm{V}})\delta ({r}_{{\rm{CI}}}-A({x}_{V},{x}_{I}))+\mathrm{(1}-{\rm{P}}({\rm{V}}))\delta ({r}_{{\rm{CI}}}-B({x}_{V},{x}_{I}))$$

where

$$\begin{array}{rcl}A({x}_{V},{x}_{I}) & = & {\rm{P}}({\rm{C}}|{x}_{V},{x}_{I}){r}_{{\rm{FF}}}({x}_{V},{x}_{I})+(1-P({\rm{C}}|{x}_{V},{x}_{I}))\,{\alpha }_{V}{x}_{V}\\ B({x}_{V},{x}_{I}) & = & {\rm{P}}({\rm{C}}|{x}_{V},{x}_{I}){r}_{{\rm{FF}}}({x}_{V},{x}_{I})+(1-P({\rm{C}}|{x}_{V},{x}_{I}))\,{\alpha }_{I}{x}_{I}.\end{array}$$

By substituting the expression for *r*_FF_ (16) into the equations above and doing some transformations we obtain

$$\begin{array}{rcl}A({x}_{V},{x}_{I}) & = & {\rm{P}}({\rm{C}}|{x}_{V},{x}_{I})\,({\alpha }_{0V}{x}_{V}+{\alpha }_{0I}{x}_{I}-{\alpha }_{V}{x}_{V})+{\alpha }_{V}{x}_{V}\\ B({x}_{V},{x}_{I}) & = & {\rm{P}}({\rm{C}}|{x}_{V},{x}_{I})\,({\alpha }_{0V}{x}_{V}+{\alpha }_{0I}{x}_{I}-{\alpha }_{I}{x}_{I})+{\alpha }_{I}{x}_{I}.\end{array}$$

By replacing the final tilt estimate generating model P(*r*|*x*_*V*_, *x*_*I*_) in (5) with (27), we can obtain the likelihood function for *r* given the stimuli. However, due to the P(C|*x*_*V*_, *x*_*I*_) expression, the integral in (5) cannot be represented in a closed form. To resolve this issue, we linearize *A*(*x*_*V*_, *x*_*I*_) and *B*(*x*_*V*_, *x*_*I*_) at *x*_*V*_ = *x*_*V*0_ = *β*_*V*_*θ*_*V*_, *x*_*I*_ = *x*_*I*0_ = *β*_*I*_*θ*_*I*_. For *A*, *B*, we obtain

28 $$A({x}_{V},{x}_{I})\approx \mathop{\underbrace{A({x}_{V0},{x}_{I0})}}\limits_{{A}_{0}({\theta }_{V},{\theta }_{I})}+\mathop{\underbrace{\frac{{\rm{d}}A}{{\rm{d}}{x}_{V}}({x}_{V0},{x}_{I0})}}\limits_{{a}_{V}({\theta }_{V},{\theta }_{I})}({x}_{V}-{x}_{V0})+\mathop{\underbrace{\frac{{\rm{d}}A}{{\rm{d}}{x}_{I}}({x}_{V0},{x}_{I0})}}\limits_{{a}_{I}({\theta }_{V},{\theta }_{I})}({x}_{I}-{x}_{I0})$$

29 $$B({x}_{V},{x}_{I})\approx \mathop{\underbrace{B({x}_{V0},{x}_{I0})}}\limits_{{B}_{0}({\theta }_{V},{\theta }_{I})}+\mathop{\underbrace{\frac{{\rm{d}}B}{{\rm{d}}{x}_{V}}({x}_{V0},{x}_{I0})}}\limits_{{b}_{V}({\theta }_{V},{\theta }_{I})}({x}_{V}-{x}_{V0})+\mathop{\underbrace{\frac{{\rm{d}}B}{{\rm{d}}{x}_{I}}({x}_{V0},{x}_{I0})}}\limits_{{b}_{I}({\theta }_{V},{\theta }_{I})}({x}_{I}-{x}_{I0}\mathrm{)}.$$

If we use the approximations for *A*(*x*_*V*_, *x*_*I*_), *B*(*x*_*V*_, *x*_*I*_) in (27), we can solve the integrals in (5). This yields

30 $$\begin{array}{rcl}{\rm{P}}({r}_{{\rm{CI}}}|{\theta }_{V},{\theta }_{I}) & = & {\rm{P}}({\rm{V}})\,(\frac{1}{\sqrt{2\pi {v}_{V}}})\,\exp \,(-\frac{{(r-{A}_{0}({\theta }_{V},{\theta }_{I}))}^{2}}{2{\sigma }_{A}^{2}})\end{array}$$

31 $$\begin{array}{lll} &  & +\mathrm{(1}-{\rm{P}}({\rm{V}}))\,(\frac{1}{\sqrt{2\pi {v}_{I}}})\,\exp \,(-\frac{{(r-{B}_{0}({\theta }_{V},{\theta }_{I}))}^{2}}{2{\sigma }_{B}^{2}})\end{array}$$

with $${\sigma }_{A}^{2}={a}_{V}^{2}{\sigma }_{V}^{2}+{a}_{I}^{2}{\sigma }_{I}^{2}$$ and $${\sigma }_{B}^{2}={b}_{V}^{2}{\sigma }_{V}^{2}+{b}_{I}^{2}{\sigma }_{I}^{2}$$.

We validated the linear approximation by comparing the results with those of performing numerical integrations for the initial few participants.

### Data analysis

The parameters to account for distortions in perception (*β*_*V*_,*β*_*I*_); the standard deviations of the sensory estimates (*σ*_*V*_,*σ*_*I*_); and the mixture and ‘tolerance for discrepancies’ prior parameters (P(V), P(C)) were treated as free parameters, resulting in a total of two to six free parameters, depending on the model. The standard deviation of the idiotropic prior *σ*_0_ was initially also included as a free parameter. However, doing so was found to result in problems with optimization convergence and generally yielded extremely large values for this parameter, suggesting that its effect on the perception of verticality was negligible. The existence of an idiotropic vector was proposed to account for biases in the SVV^8^, but the findings of previous studies found this prior not to affect the SHV^35,36^. Consequently, we fixed the value of *σ*_0_ to 100. This renders the prior effectively uninformative, but we chose to include it for consistency with the literature.

We fitted the CC and FF models by minimizing the negative log-likelihood of the tilt estimates (*r* = −*R*) given the model $-{{\rm{\Sigma }}}_{i=1}^{n}\,\mathrm{log}\,({\rm{\Pr }}({r}_{{{\rm{model}}}_{i}}|{\theta }_{{V}_{i}},{\theta }_{{I}_{i}}))$, using the fmincon routine in MATLAB. The fmincon routine was not suitable to fit the SS and CI models: these are mixture models, and directly maximizing the likelihood can lead to numerical issues. We therefore applied the Expectation-Maximization (EM) algorithm to fit these models^37^. In the EM-algorithm, membership of mixture components is treated as a latent variable. The model likelihood is maximized iteratively, by repeating a set of two steps: first, the probability of each observation belonging to either component of the mixture is determined given an initial set of parameters. Second, the model parameters are re-estimated, while taking the probability of component membership calculated in the previous step into account. To re-estimate the parameters in the second step, we again minimized the model negative log-likelihoods using the MATLAB fmincon routine. The iterations were terminated when the change in model likelihood was smaller than 1 × 10^−6^.

To determine which model best approximated participant responses, we compared model Bayesian Information Criterion (BIC) scores^38^. The BIC is a penalized likelihood score, taking into account the number of observations and the number of free parameters in each model. The model with the lowest BIC score is considered the best in an absolute sense. Differences in model BIC scores (ΔBIC) between 0–2; 2–6; 6–10 are considered negligible, positive, and strong evidence, respectively, and ΔBIC > 10 are considered decisive evidence.

For each of the models, we evaluated the fit of an additional version, where the values of the *β*_*V*_ and *β*_*I*_ parameters were fixed at a value of 1, reflecting an assumption that perception itself is veridical. Here, distortion of the final response *R* was attributed to an over- or underestimation of the angle of the rod. This was implemented as a linear transformation of the response random variable: *R* = *β*_*r*_*r*_model_, with ${\rm{Var}}(R)={\beta }_{r}^{2}\,{\rm{Var}}\,({r}_{{\rm{model}}})$. In this version, the scaling parameter (*β*_*r*_) affects the noise parameters (*σ*_*m*_). To illustrate, when the response reflects a consistent underestimation of the tilt angle, this will result in *β*_*r*_ < 1. This also implies that the noise parameter for perceived tilt *r* must be increased to fit the variability in responses *R*, compared to the first version of the models. Ultimately, this version of the models was found to provide a reasonable alternative only for the FF model, and we therefore chose not to further consider the findings of this version of the models. The model fits are available as supplementary material, in Tables S8–S14.

It should also be noted that while it is theoretically possible that distortions are introduced both at the perceptual and response levels, it was not possible to estimate both effects simultaneously because distortion parameters at both levels allow similar behavior, resulting in an infinite number of equivalent solutions.

## Results

In the following, we separately present the findings on model fits and parameter estimates. As an illustration of the findings, Fig. 2 shows data and model fits for an example participant. Figures showing the data of all individual participants are provided as supplementary material Figs S1–S36.

**Figure 2:**
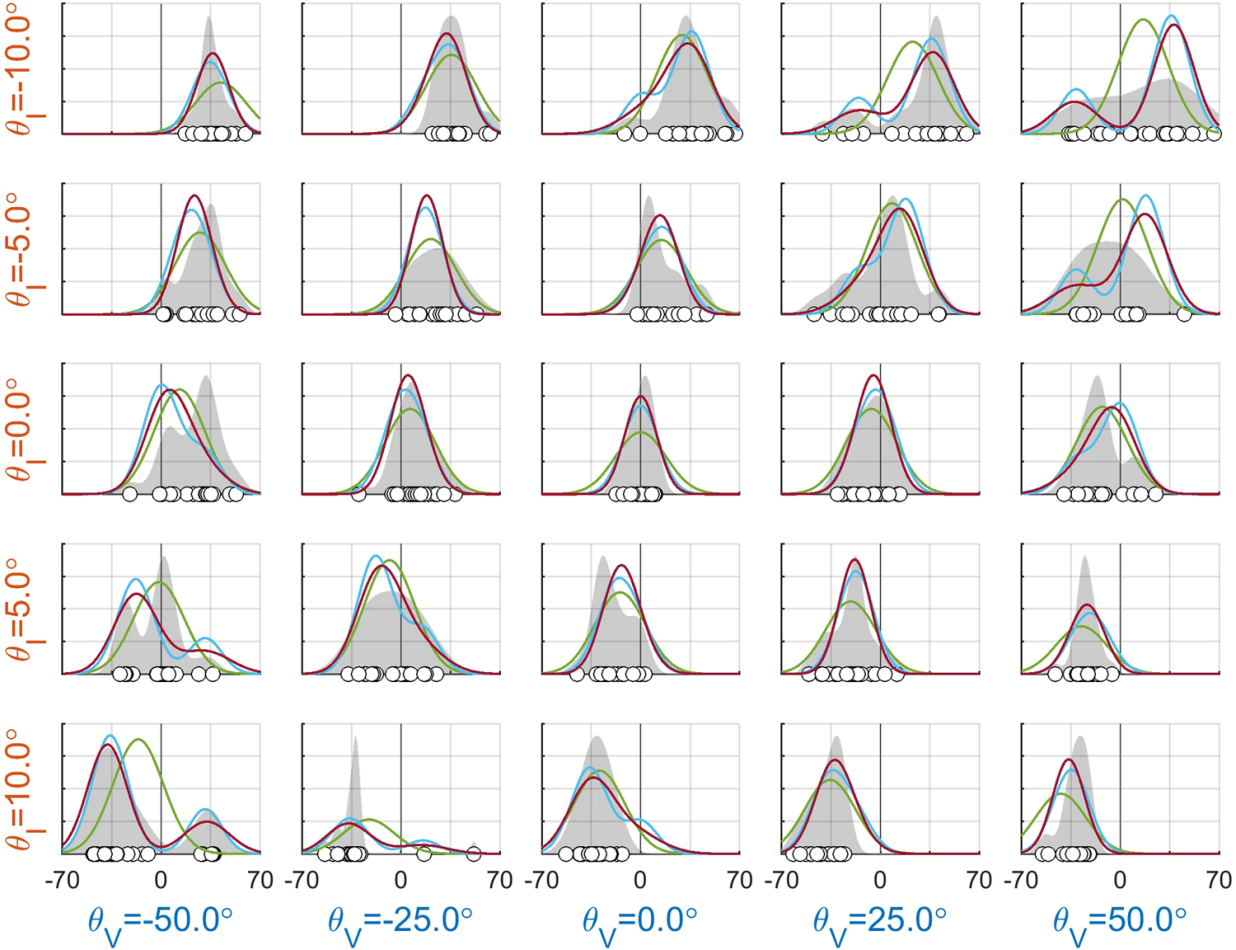
Overview of the results for an example participant (31). Each panel shows the data of a particular experimental condition. Responses (white dots) reflect the negative of the perceived tilt. The gray-shaded areas show the corresponding kernel density estimates. The thin black line at 0° is the Earth-vertical. The colored lines represent the response densities according to the SS (blue), FF (green), and CI (red) models that allowed for distortion in perceptions. Note how the CI model allows for behaviors in between the FF and SS models.

### Model comparisons

In the first iteration of the experiment, where stimuli with discrepancies up to ±20° were presented, the evidence did not allow to decide upon a best fitting model, as the results were tied between the FF model (BIC = 44710.67) and the CI model (BIC = 44710.39), resulting in an overall ΔBIC of 0.28, which is considered negligible evidence. Inspection of individual results also revealed a considerable variability between participants with respect to the preferred model: the CC_I_ model provided the best fit in six cases; the SS model in one case; the FF model in seven; and the CI model in the five remaining cases.

In the second iteration, where stimuli with discrepancies up to ±26° were presented, the evidence favored the CI model, as indicated by a ΔBIC score of 16.33. The individual results however again exhibited variability between participants. Here, the SS model was preferred in one case, the FF model in three, and the CI model in five cases.

The results of the third iteration of the experiment, with discrepancies up to ±60°, favored the CI model. This was evidenced by a ΔBIC value of 268.46, compared to the runner-up model SS. Individual results were also consistent, providing support for the CI model in seven out of eight cases, and providing support for the SS model in the remaining case (ΔBIC = 4.42, compared to CI). Overall ΔBIC scores for the three iterations are presented in Table 1. The negative log-likelihood and BIC scores obtained for individual participants are presented in supplementary material Tables S1 and S2.

**Table 1:** Overall ΔBIC scores for the three iterations of the experiment.

Iteration	ΔBIC				
	CC_V_	CC_I_	SS	FF	CI
1	9358.77	478.37	363.75	0.28	0
2	2951.60	278.28	142.63	16.33	0
3	3590.52	2314.98	268.46	426.58	0

ΔBIC values are calculated as the difference between the BIC score obtained for the model of the corresponding column and the best fitting model. Overall BIC scores were calculated using the sums (over participants) of the model log-likelihoods, the number of parameters, and the number of observations.

### Parameter estimates

Summaries of the obtained estimates for the scaling parameters *β*_*V*_,*β*_*I*_, the standard deviations of the sensory estimates *σ*_*V*_ and *σ*_*I*_, and the mixture and prior parameters P(V) and P(C) for each of the three iterations of the experiment are presented in Table 2. The obtained parameter estimates for each model/individual are available as supplementary material Tables S3–S7.

**Table 2:** Median parameter values over participants (and standard deviation).

Parameter	Model					
	CC_V_	CC_I_	SS	FF	CI	bounds
**Iteration 1**						
*β*_*V*_	0.12 (0.15)		0.57 (0.47)	0.52 (0.66)	0.82 (1.54)	[−5:5]
*σ*_*V*_	7.60 (4.39)		4.59 (5.08)	8.13 (6.45)	7.65 (8.83)	[1:∞]
*β*_*I*_		0.86 (0.59)	0.97 (0.57)	1.35 (0.69)	1.25 (0.94)	[−5:5]
*σ*_*I*_		4.37 (1.82)	3.97 (1.66)	5.13 (2.43)	4.12 (2.32)	[1:∞]
P(V)			0.03 (0.14)		0.50 (0.41)	[0:1]
P(C)					0.82 (0.39)	[0:1]
**Iteration 2**						
*β*_*V*_	0.14 (0.18)		0.36 (0.85)	0.43 (0.35)	0.77 (1.92)	[−5:5]
*σ*_*V*_	8.35 (3.33)		2.99 (3.49)	8.91 (3.59)	10.98 (8.25)	[1:∞]
*β*_*I*_		0.69 (0.37)	0.80 (0.36)	1.12 (0.49)	1.09 (0.40)	[−5:5]
*σ*_*I*_		5.84 (2.44)	5.23 (1.40)	7.47 (3.80)	5.43 (1.65)	[1:∞]
P(V)			0.07 (0.24)		0.50 (0.39)	[0:1]
P(C)					0.27 (0.37)	[0:1]
**Iteration 3**						
*β*_*V*_	0.22 (0.18)		0.58 (0.13)	0.77 (0.56)	0.66 (0.71)	[−5:5]
*σ*_*V*_	13.91 (6.43)		8.56 (3.26)	17.63 (13.53)	10.94 (8.37)	[1:∞]
*β*_*I*_		1.46 (0.89)	1.98 (0.74)	2.17 (0.71)	2.68 (1.17)	[−5:5]
*σ*_*I*_		15.36 (5.68)	8.04 (2.78)	14.89 (5.33)	12.87 (7.72)	[1:∞]
P(V)			0.26 (0.32)		0.21 (0.36)	[0:1]
P(C)					0.05 (0.32)	[0:1]

Values are split per experiment iteration. The lower bounds of 1 for *σ*_*V*_, *σ*_*I*_ were chosen to prevent cases where fitting of the mixture models would result in explanation of a single outlier with a dedicated component with near-zero standard deviation.

The median value of scaling parameter *β*_*V*_ varied considerably between models and iterations, ranging from 0.12 (CC_V_, iteration 1) to 0.82 (CI, iteration 1). For the CC_V_ model, the parameter’s value can be interpreted as a regression coefficient, and as such indicates a minor effect of vision on tilt estimates. For the latter models, the parameter’s overall median value was 0.57. A value below one indicates that the visual tilt was underestimated. The median value for the visual noise parameter *σ*_*V*_ ranged from 2.99 (SS, iteration 2) to 17.63 (FF, iteration 3), and was largest in the third iteration.

The median value for scaling parameter *β*_*I*_ showed a similar variability, ranging from 0.69 (CC_I_, iteration 2) to 2.68 (CI, iteration 3), but was generally larger than 1 (overall median value 1.33). For the CC_I_ model, the parameter can again be interpreted as a regression coefficient, and indicates a larger effect for physical tilt than for visual tilt. The finding that the parameter’s value was generally larger than one for the SS, FF, and CI models indicates that physical tilt was overestimated. The median value for the inertial noise parameter *σ*_*I*_ ranged from 3.97 to 15.36, and was also the largest in the third iteration.

Assuming forced fusion, the observed values of the *σ*_*V*_,*σ*_*I*_ parameters can be translated into relative contributions of visual and inertial information to an integrated estimate. For the FF model, the observed values translate into visual:inertial weights of 0.28:0.72, 0.41:0.59, and 0.42:0.58, for the three iterations, respectively; for the CI model, these weights would correspond to 0.23:0.77, 0.20:0.80, and 0.59:0.41.

Parameter P(V) was generally close to zero for the SS model (medians 0.03, 0.07, 0.26), suggesting that participants generally relied on inertial information (as in the CC_I_ model), but occasionally lapsed by relying on visual information. In the CI model, the value of this parameter was larger in the first two iterations (medians 0.50, 0.50), indicating that responses were more likely to reflect visual information when a discrepancy was likely, but closer to the SS model estimate in the final iteration (0.21).

The a-priori belief that signals will have a common cause, reflected by parameter P(C) of the CI model had median values of 0.82, 0.27, 0.05, suggesting that the range of discrepancies affects participants′ a-priori tendency to assume a common cause.

## Discussion

We investigated how perceptions of upright are constructed under conditions of uncertainty about the veracity of visual and inertial cues. We manipulated the maximum discrepancy between the orientation suggested by these cues in three experimental iterations, with maximum discrepancies increasing from ±20° in the first iteration, to ±26° in the second, and ±60° in the final iteration.

### Perceptual bias

The perception of verticality has been shown to be subject to a number of biases. Most notably, distortions are caused by ocular counterrolling (OCR), and hysteresis.

OCR is roll rotation of the eyes in the direction opposite to the inducing stimulus. OCR can be induced by physical as well as visual tilt stimuli (e.g.^39–42^), and can amount to up to approximately 10% of the roll-stimulus angle^2,42^. It has been shown that the perceptual system does not correct for such torsional motion^43^. Our setup did not allow us to measure OCR directly. Instead, we addressed the possibility of systematic distortions by including scaling parameters for the unisensory visual and inertial tilt estimates in the modeling. The visual scaling parameter was found to be generally below 1, which is consistent with the findings on OCR discussed above, although the contribution of the visual information was scaled down more than the 10% expected from the literature. In contrast, the inertial scaling parameter was generally considerably larger than 1, indicating physical tilt was overestimated. This finding appears to be consistent the E-effect, which is an apparent overestimation of head tilt for (relatively) small physical tilt angles^2,44,45^. It is nevertheless surprising to note that the observed overestimations, and subsequently the scaling parameters for physical tilt (*β*_*I*_), were considerably larger in the third iteration of the experiment than in the first iteration of the experiment, whereas the presented physical tilt angles were equal. Because the only difference in the paradigm between these iterations was an increase in the range of visual tilt angles, we speculate that the range of visually perceived angles may affect expectations on the range of physical tilt angles, and consequently affect the scaling of inertial sensory estimates. This could be interpreted as a cross-modal range effect (see e.g.^46^).

Hysteresis is the phenomenon where the state of a system depends on its previous state. It has been shown that the SHV task is subject to this effect^36,47^. We tested three different methods of transitioning between stimuli in the first iteration of the experiment to address this effect. For the first method, the camera was turned off during transitions; for the second method, heave and sway vibrations were added to the transitional rotations; and for the third method, the camera was always kept on whereas the vibrations were omitted. The first allows hysteresis effects of the inertial cue, as this cue is always present and could be tracked during transitions. Due to the vibrations, this is not feasible for the second method. Both visual and inertial hysteresis effects are possible for the third method, but the senses present conflicting information because stimuli were presented in random order, with the added requirement that there was always transitional rotation. Ultimately, the manipulations did not appear to affect the results, suggesting that participants were able to evaluate the stimuli independently. We chose the latter method for the remainder of the experiment because the sensory modalities are treated in the most similar way.

### Model comparisons

The results of the first and second iterations did not allow us to discriminate between the models with certainty, as the overall evidence was tied between the FF and CI models. On an individual level, the FF and CI models provided the best explanation of the data for an equal number participants. Preference of the FF model over the CI model would imply that the SHV is constructed by mandatory fusion of multisensory information on spatial orientation. The standard deviations of visual and inertial estimates further indicated that the contribution of the visual cue to the perceived upright was smaller than the contribution of the inertial cue, with approximate relative weights of 0.35:0.65 (visual:inertial). The observed relative weightings resemble those reported in studies on the SVV^14,18^ and SHV^45^, and the consistency of responses with predictions from FF has also been reported by studies assessing the perceptual upright with other methods, such as the Oriented Character Recognition Test (OCHART)^14,18^; the SVV^36^; and SBT^17^.

Despite these parallels, a conclusion that perceptions of upright are constructed according to FF would be at odds with recent findings on multisensory interactions regarding perception of other environmental properties, such as audio-visual localization tasks (e.g.^22–25^) and visual-inertial heading estimation^26,27^, where it was found that the CNS includes assessments of signal causality in the formation of perceptions. Evaluation of the findings of the first two iterations indicated that predictions made by the different models were quite similar, making it difficult to discriminate between them even for the largest discrepancies. To address this, we used the CI parameter estimates obtained in the first and second iteration of the experiment to simulate response distributions, and to determine whether these would diverge more clearly, for even larger discrepancies. Based on the findings of these simulations, we increased the maximum discrepancy to ±60°, and performed a third iteration of the experiment with eight additional participants. This had the desired effect, as here the models produced distinct predictions. Evaluation of the responses further provided decisive evidence in favor of the CI model. Because CI behaves as FF when discrepancies are small (i.e., when the size of the discrepancies does not clearly exceed the respective sensory noises), the additional findings do not conflict with the cases where the FF model was preferred in the first two iterations of the experiment; FF was the preferred model because it has fewer parameters. We conclude that, consistent with the hypothesis, the CNS incorporates assessments of signal causality in the perception of verticality, but that this effect becomes unambiguous only for large discrepancies.

## Electronic supplementary material

Supplementary Information

Dataset 1

## References

[1] Howard IP, Bergström SS, Ohmi M (1990). Shape from shading in different frames of reference. Percept..

[2] Howard, I. P. *Human visual orientation* (John Wiley & Sons, 1982).

[3] Lackner, J. R. & Graybiel, A. Postural illusions experienced during z-axis recumbent rotation and their dependence upon somatosensory stimulation of the body surface. *Aviat*. *space*, *environmental medicine* (1978). 637808

[4] Lackner, J. R. & Graybiel, A. Some influences of touch and pressure cues on human spatial orientation. *Aviat*. *space*, *environmental medicine* (1978). 656007

[5] Vaitl D, Mittelstaedt H, Baisch F (1997). Shifts in blood volume alter the perception of posture. Int. J. Psychophysiol..

[6] Vaitl D, Mittelstaedt H, Saborowski R, Stark R, Baisch F (2002). Shifts in blood volume alter the perception of posture: further evidence for somatic graviception. Int. J. Psychophysiol..

[7] Mittelstaedt H (1996). Somatic graviception. Biol. psychology.

[8] Mittelstaedt H (1983). A new solution to the problem of the subjective vertical. Naturwissenschaften.

[9] Clark, J. J. & Yuille, A. L. *Data fusion for sensory information processing systems*, vol. 105 (Springer Science & Business Media, 1990).

[10] Landy MS, Maloney LT, Johnston EB, Young M (1995). Measurement and modeling of depth cue combination: in defense of weak fusion. Vis. research.

[11] Ernst MO, Banks MS (2002). Humans integrate visual and haptic information in a statistically optimal fashion. Nature.

[12] Hillis JM, Ernst MO, Banks MS, Landy MS (2002). Combining sensory information: mandatory fusion within, but not between, senses. Sci..

[13] Ernst MO, Bülthoff HH (2004). Merging the senses into a robust percept. Trends in cognitive sciences.

[14] Dyde RT, Jenkin MR, Harris LR (2006). The subjective visual vertical and the perceptual upright. Exp. Brain Res..

[15] MacNeilage PR, Banks MS, Berger DR, Bülthoff HH (2007). A bayesian model of the disambiguation of gravitoinertial force by visual cues. Exp. Brain Res..

[16] Vingerhoets RAA, De Vrijer M, Van Gisbergen JA, Medendorp WP (2009). Fusion of visual and vestibular tilt cues in the perception of visual vertical. J. neurophysiol..

[17] Clemens IA, De Vrijer M, Selen LP, Van Gisbergen JA, Medendorp WP (2011). Multisensory processing in spatial orientation: an inverse probabilistic approach. J. Neurosci..

[18] Harris LR, Jenkin M, Jenkin H, Zacher JE, Dyde RT (2017). The effect of long-term exposure to microgravity on the perception of upright. npj Microgravity.

[19] Dyde RT, Jenkin MR, Jenkin HL, Zacher JE, Harris LR (2009). The effect of altered gravity states on the perception of orientation. Exp. brain research.

[20] Kaptein RG, Van Gisbergen JA (2004). Interpretation of a discontinuity in the sense of verticality at large body tilt. J. Neurophysiol..

[21] de Winkel KN, Clément G, Groen EL, Werkhoven PJ (2012). The perception of verticality in lunar and martian gravity conditions. Neurosci. letters.

[22] Körding KP (2007). Causal inference in multisensory perception. PLoS One.

[23] Sato Y, Toyoizumi T, Aihara K (2007). Bayesian inference explains perception of unity and ventriloquism aftereffect: identification of common sources of audiovisual stimuli. Neural computation.

[24] Beierholm, U., Shams, L., Ma, W. J. & Koerding, K. Comparing bayesian models for multisensory cue combination without mandatory integration. In *Advances in neural information processing systems*, 81–88 (2008).

[25] Wozny DR, Beierholm UR, Shams L (2010). Probability matching as a computational strategy used in perception. PLoS Comput. Biol..

[26] De Winkel KN, Katliar M, Bülthoff HH (2017). Causal inference in multisensory heading estimation. PloS one.

[27] Acerbi, L., Dokka, K., Angelaki, D. E. & Ma, W. J. Bayesian comparison of explicit and implicit causal inference strategies in multisensory heading perception. *bioRxiv* 150052 (2017). 10.1371/journal.pcbi.1006110 PMC6063401 30052625

[28] Harris, L. R., Jenkin, M., Dyde, R. T. & Jenkin, H. Enhancing visual cues to orientation: Suggestions for space travelers and the elderly. In *Progress in brain research*, vol. 191, 133–142 (Elsevier, 2011). 10.1016/B978-0-444-53752-2.00008-4 21741549

[29] Murray RF, Morgenstern Y (2010). Cue combination on the circle and the sphere. J. vision.

[30] De Winkel KN, Katliar M, Bülthoff HH (2015). Forced fusion in multisensory heading estimation. PLoS One.

[31] Bhattacharyya A (1943). On a measure of divergence between two statistical populations defined by their probability distributions. Bull. Calcutta Math. Soc..

[32] Rock I, Victor J (1964). Vision and touch: An experimentally created conflict between the two senses. Sci..

[33] de Winkel KN, Weesie J, Werkhoven PJ, Groen EL (2010). Integration of visual and inertial cues in perceived heading of self-motion. J. vision.

[34] De Winkel K (2013). Integration of visual and inertial cues in the perception of angular self-motion. Exp. brain research.

[35] Bortolami SB, Pierobon A, DiZio P, Lackner JR (2006). Localization of the subjective vertical during roll, pitch, and recumbent yaw body tilt. Exp. brain research.

[36] Vingerhoets RAA, Medendorp WP, Van Gisbergen JA (2008). Body-tilt and visual verticality perception during multiple cycles of roll rotation. J. neurophysiology.

[37] Dempster, A. P., Laird, N. M. & Rubin, D. B. Maximum likelihood from incomplete data via the em algorithm. *J*. *royal statistical society*. *Ser*. *B* (*methodological*) 1–38 (1977).

[38] Schwarz G (1978). Estimating the dimension of a model. The annals statistics.

[39] Miller EF (1962). Counterrolling of the human eyes produced by head tilt with respect to gravity. Acta oto-laryngologica.

[40] Cheung B (1992). Human ocular torsion during parabolic flights: an analysis with scleral search coil. Exp. brain research.

[41] Kingma H, Stegeman P, Vogels R (1997). Ocular torsion induced by static and dynamic visual stimulation and static whole body roll. Eur. Arc. Oto-rhino-laryngology.

[42] Bockisch CJ, Haslwanter T (2001). Three-dimensional eye position during static roll and pitch in humans. Vis. research.

[43] Wade SW, Curthoys IS (1997). The effect of ocular torsional position on perception of the roll-tilt of visual stimuli. Vis. research.

[44] Müller, G. E. *Über das Aubertsche Phänomen* (JA Barth, 1915).

[45] Barnett-Cowan M, Harris LR (2008). Perceived self-orientation in allocentric and egocentric space: effects of visual and physical tilt on saccadic and tactile measures. Brain research.

[46] Petzschner FH, Glasauer S (2011). Iterative bayesian estimation as an explanation for range and regression effects: a study on human path integration. J. Neuroscience.

[47] Schuler JR, Bockisch CJ, Straumann D, Tarnutzer AA (2010). Precision and accuracy of the subjective haptic vertical in the roll plane. BMC neuroscience.

