# Supplementary material for: Causal Inference in the Perception of Verticality
Source: Sci Rep. 2018 Apr 3;8:5483. doi: 10.1038/s41598-018-23838-w (PMC5882842; doi:10.1038/s41598-018-23838-w)

Supplementary Information

Causal Inference in the Perception of Verticality

Ksander N. de Winkel[1,*], Mikhail Katliar[1], Daniel Diers[1], Heinrich H. Bülthoff[1]

[1]Department of Human Perception, Cognition, and Action, Max Planck Institute for Biological Cybernetics, Max-Planck-Ring 8, 72076, Tübingen, Germany

[*]ksander.dewinkel@tuebingen.mpg.de

Supplementary material Table S1. Biased perception models negative log-likelihood scores (nLL). Lowest values are boldfaced.

	nLL					
pp	CC_V	CC_I	FF	SS	CI	n_obs
1	1518.84	1079.15	1078.04	1078.20	**1077.57**	400
2	1522.41	1175.79	1170.87	1175.79	**1170.86**	400
3	1534.42	1389.39	1388.96	**1364.67**	1364.67	400
4	1423.24	1105.29	1100.58	1103.95	**1100.44**	400
5	1846.92	1486.15	1485.57	1486.15	**1485.38**	400
6	1439.28	1288.42	1245.27	1252.20	**1237.59**	400
7	1603.13	1330.90	1330.89	1330.86	**1328.65**	399
8	1250.84	1056.19	1044.52	1029.78	**1015.94**	400
9	1310.38	1072.59	1034.11	1071.57	**1033.36**	400
10	1527.55	1083.11	1081.69	1083.11	**1080.94**	400
11	1266.30	1219.17	1149.07	1126.14	**1110.55**	375
12	1338.58	1156.83	1154.43	1151.47	**1148.57**	400
13	1296.54	1059.71	1032.45	1059.71	**1031.87**	398
14	1335.17	1037.91	1012.01	1037.91	**1012.01**	400
15	1405.66	1229.19	1229.16	1225.94	**1225.11**	400
16	1353.24	1337.22	1277.60	1296.83	**1274.72**	400
17	1240.27	1033.88	1001.65	1018.66	**989.10**	400
18	1377.62	1217.58	1177.91	1208.61	**1177.60**	400
19	1309.40	1101.11	1090.97	1098.53	**1085.90**	400
sum	26899.79	22459.59	22085.75	22200.09	**21950.82**	7572
20	1548.60	1247.50	1247.20	1230.80	**1227.48**	400
21	1415.17	1251.83	1240.76	1247.62	**1240.66**	400
22	1560.87	1542.47	1526.81	**1522.88**	1522.88	399
23	1654.08	1371.99	1368.61	1370.88	**1362.43**	399
24	1224.84	1078.54	1035.88	1076.90	**1034.71**	400
25	1408.15	1148.26	1138.88	1144.44	**1127.69**	400
26	1390.30	1269.31	1241.60	1245.87	**1230.52**	399
27	1554.60	1453.13	1440.43	1436.86	**1432.39**	394
28	1226.87	1283.82	1218.57	1217.06	**1214.67**	400
sum	12983.50	11646.84	11458.73	11493.31	**11393.43**	3591
29	2456.62	2232.92	2193.99	2188.25	**2161.37**	525
30	2141.72	2373.67	2091.33	2078.56	**2056.95**	525
31	2481.66	2319.81	2259.24	2206.94	**2202.10**	525
32	2043.16	1754.18	1753.59	1728.85	**1728.85**	525
33	2260.48	1900.51	1895.13	1870.98	**1853.71**	525
34	2088.67	2104.68	1958.55	1923.48	**1901.05**	525
35	1992.39	2314.09	1963.47	1975.98	**1956.84**	525
36	2101.12	1928.20	1816.49	1853.69	**1805.60**	525
sum	17565.82	16928.05	15931.81	15826.73	**15666.48**	4200
overall	57449.11	51034.48	49476.30	49520.13	**49010.73**	15363

Supplementary material Table S2. Biased perception models Bayesian Information Criterion scores (BIC). Best model scores are boldfaced.

	BIC				
pp	CC_V	CC_I	FF	SS	CI
1	3045.98	**2166.61**	2172.70	2177.16	2180.06
2	3053.12	2359.88	**2358.36**	2372.34	2366.64
3	3077.15	2787.09	2794.53	**2750.10**	2754.26
4	2854.78	2218.88	**2217.78**	2228.67	2225.80
5	3702.16	**2980.62**	2987.75	2993.08	2995.68
6	2886.86	2585.15	2507.15	2525.18	**2500.11**
7	3214.56	**2670.09**	2678.38	2682.48	2682.22
8	2509.98	2120.68	2105.66	2080.33	**2056.81**
9	2629.06	2153.48	**2084.84**	2163.91	2091.63
10	3063.40	**2174.53**	2180.00	2186.99	2186.81
11	2540.79	2446.51	2314.49	2272.72	**2245.63**
12	2685.46	**2321.97**	2325.48	2323.72	2322.06
13	2601.38	2127.72	**2081.49**	2140.17	2088.63
14	2678.64	2084.14	**2040.64**	2096.60	2048.94
15	2819.64	**2466.70**	2474.94	2472.65	2475.13
16	2714.79	2682.76	**2571.81**	2614.43	2574.37
17	2488.86	2076.06	2019.91	2058.08	**2003.12**
18	2763.55	2443.47	**2372.44**	2437.98	2380.11
19	2627.11	2210.52	2198.55	2217.83	**2196.71**
iteration 1	54069.16	45188.76	44710.67	45074.14	**44710.39**
20	3105.51	2503.31	2511.02	2482.38	**2479.89**
21	2838.64	2511.97	**2498.13**	2516.01	2506.24
22	3130.05	3093.23	3070.23	**3066.52**	3070.67
23	3316.47	2752.28	2753.81	2762.52	**2749.76**
24	2457.98	2165.39	**2088.38**	2174.56	2094.34
25	2824.62	2304.82	2294.37	2309.64	**2280.30**
26	2788.91	2546.91	2499.80	2512.50	**2485.94**
27	3117.49	2914.55	2897.41	2894.41	**2889.61**
28	2462.06	2575.94	**2453.76**	2454.89	2454.27
iteration 2	26081.27	23407.95	23146.00	23272.29	**23129.67**
29	4922.10	4474.68	4405.68	4398.62	**4349.30**
30	4292.29	4756.20	4200.37	4179.25	**4140.46**
31	4972.17	4648.47	4536.19	4436.02	**4430.76**
32	4095.17	3517.21	3524.89	**3479.83**	3484.25
33	4529.81	3809.87	3807.97	3764.09	**3733.97**
34	4186.20	4218.21	3934.81	3869.10	**3828.66**
35	3993.62	4637.03	3944.65	3974.08	**3940.23**
36	4211.09	3865.25	3650.69	3729.50	**3637.76**
iteration 3	35235.72	33960.18	32071.78	31913.66	**31645.20**

Supplementary material Table S3. Biased perception Visual Cue Capture model parameter estimates.

pp	beta_V	sigma_V
1	0.038	10.848
2	0.102	10.947
3	0.052	11.285
4	0.083	8.523
5	0.081	25.262
6	0.381	8.874
7	0.010	13.573
8	0.115	5.527
9	0.210	6.418
10	0.044	11.090
11	0.516	7.102
12	0.068	6.889
13	0.180	6.301
14	0.160	6.830
15	0.009	8.154
16	0.494	7.147
17	0.176	5.383
18	0.306	7.599
19	0.120	6.402
median	0.115	7.599
st.dev	0.149	4.393
20	-0.023	11.697
21	0.140	8.352
22	0.351	12.187
23	0.109	15.463
24	0.171	5.178
25	0.100	8.205
26	0.229	7.914
27	-0.260	12.612
28	0.345	5.205
median	0.140	8.352
st.dev	0.177	3.329
29	0.192	26.991
30	0.522	14.452
31	0.279	28.413
32	-0.009	11.940
33	0.038	18.230
34	0.251	13.039
35	0.488	10.826
36	0.161	13.357
median	0.221	13.905
st.dev	0.178	6.428

Note. Parameters were constrained to lie within the following bounds: beta_V: -5-5; sigma_V: 1-1e2; beta_I: -5-5; sigma_I: 1-1e2; PrV: 0-1; PrC: 0-1.

Supplementary material Table S4. Biased perception Inertial Cue Capture model parameter estimates.

pp	beta_I	sigma_I
1	1.440	3.595
2	1.403	4.579
3	1.147	7.827
4	1.076	3.838
5	3.198	9.988
6	0.989	6.074
7	1.648	6.814
8	0.627	3.394
9	0.785	3.537
10	1.474	3.631
11	0.700	6.260
12	0.755	4.367
13	0.766	3.470
14	0.863	3.243
15	0.882	5.235
16	0.568	6.865
17	0.635	3.210
18	0.854	5.085
19	0.738	3.798
median	0.863	4.367
st.dev	0.588	1.821
20	1.118	5.481
21	0.693	5.541
22	0.529	11.630
23	1.457	7.557
24	0.440	3.590
25	0.767	4.274
26	0.624	5.836
27	0.910	9.717
28	0.114	6.004
median	0.693	5.836
st.dev	0.369	2.443
29	3.018	17.269
30	0.889	22.822
31	3.045	20.498
32	1.377	6.853
33	2.217	9.072
34	1.164	13.449
35	0.512	20.267
36	1.537	9.568
median	1.457	15.359
st.dev	0.887	5.676

Note. Parameters were constrained to lie within the following bounds: beta_V: -5-5; sigma_V: 1-1e2; beta_I: -5-5; sigma_I: 1-1e2; PrV: 0-1; PrC: 0-1.

Supplementary material Table S5. Biased perception Switching Strategy model parameter estimates.

pp	beta_V	sigma_V	beta_I	sigma_I	PrV
1	0.438	10.386	1.443	3.484	0.008
2	0.753	9.019	1.403	4.579	0.000
3	0.831	5.969	1.398	6.323	0.171
4	0.573	1.000	1.084	3.759	0.012
5	0.189	23.321	3.198	9.988	0.000
6	0.570	3.794	1.249	4.669	0.257
7	0.118	11.848	1.651	6.774	0.003
8	0.639	3.276	0.678	2.801	0.112
9	0.067	1.006	0.801	3.473	0.020
10	-1.230	9.287	1.474	3.631	0.000
11	0.801	3.466	1.087	3.967	0.511
12	0.235	8.643	0.772	4.109	0.026
13	0.501	2.798	0.766	3.470	0.000
14	0.857	4.585	0.863	3.243	0.000
15	0.243	4.574	0.967	4.848	0.097
16	0.810	5.664	0.810	5.175	0.410
17	0.567	3.502	0.666	2.778	0.098
18	0.835	4.585	0.897	4.547	0.108
19	0.397	1.504	0.761	3.676	0.030
median	0.567	4.585	0.967	3.967	0.026
st.dev	0.466	5.083	0.566	1.658	0.143
20	-1.928	2.132	1.125	5.038	0.020
21	0.114	1.467	0.750	5.379	0.069
22	0.519	11.517	1.155	6.463	0.627
23	0.357	3.953	1.481	7.373	0.018
24	0.832	2.749	0.426	3.387	0.051
25	0.103	2.991	0.803	4.026	0.039
26	0.712	2.684	0.703	5.146	0.108
27	-0.980	10.575	1.040	8.001	0.174
28	0.509	4.147	0.246	5.231	0.619
median	0.357	2.991	0.803	5.231	0.069
st.dev	0.853	3.491	0.362	1.403	0.235
29	0.699	11.507	3.212	13.257	0.220
30	0.617	9.819	1.888	12.532	0.770
31	0.631	11.775	3.678	12.997	0.255
32	0.724	1.000	1.372	6.362	0.011
33	0.532	7.351	2.252	7.998	0.045
34	0.416	6.450	1.720	8.032	0.576
35	0.503	9.739	2.066	8.042	0.935
36	0.321	7.374	1.754	6.326	0.265
median	0.575	8.557	1.977	8.037	0.260
st.dev	0.131	3.259	0.744	2.783	0.317

Note. Parameters were constrained to lie within the following bounds: beta_V: -5-5; sigma_V: 1-1e2; beta_I: -5-5; sigma_I: 1-1e2; PrV: 0-1; PrC: 0-1.

Supplementary material Table S6. Biased perception Forced Fusion model parameter estimates.

pp	beta_V	sigma_V	beta_I	sigma_I
1	1.779	24.597	1.472	3.626
2	0.455	9.610	1.803	5.134
3	0.103	11.071	2.303	11.112
4	1.983	18.570	1.123	3.878
5	0.428	23.673	3.897	11.065
6	0.833	8.093	1.813	7.394
7	0.018	11.712	2.497	8.407
8	0.360	5.853	0.919	3.993
9	0.577	5.333	1.232	4.027
10	1.934	24.220	1.508	3.662
11	0.994	7.376	1.358	7.322
12	0.165	6.791	1.279	5.654
13	0.517	5.561	1.159	3.990
14	2.111	11.048	0.934	3.163
15	0.025	8.594	1.405	6.615
16	0.930	8.127	1.210	8.645
17	0.496	4.985	0.982	3.684
18	0.828	7.590	1.352	5.801
19	0.354	6.379	1.113	4.552
median	0.517	8.127	1.352	5.134
st.dev	0.664	6.451	0.693	2.432
20	-0.106	11.802	1.427	6.195
21	0.381	8.914	1.094	6.782
22	0.661	15.476	1.119	16.377
23	0.453	15.481	1.905	8.594
24	0.411	5.003	0.754	4.225
25	0.428	8.658	0.999	4.771
26	0.490	7.976	1.170	7.467
27	-0.511	13.127	1.893	13.611
28	0.570	6.562	0.289	8.122
median	0.428	8.914	1.119	7.467
st.dev	0.353	3.589	0.485	3.798
29	1.531	46.856	3.417	17.285
30	0.801	16.416	2.449	22.310
31	1.700	46.730	3.584	20.153
32	-0.037	13.767	1.832	7.915
33	0.140	17.599	3.005	10.497
34	0.747	17.659	1.733	12.487
35	0.665	12.019	1.864	19.941
36	0.829	17.650	1.898	8.619
median	0.774	17.625	2.173	14.886
st.dev	0.559	13.532	0.713	5.327

Note. Parameters were constrained to lie within the following bounds: beta_V: -5-5; sigma_V: 1-1e2; beta_I: -5-5; sigma_I: 1-1e2; PrV: 0-1; PrC: 0-1.

Supplementary material Table S7. Biased perception Causal Inference model parameter estimates.

pp	beta_V	sigma_V	beta_I	sigma_I	PrV	PrC
1	0.127	7.301	1.918	4.091	0.999	0.949
2	0.998	14.324	1.561	4.772	0.500	0.963
3	0.831	5.975	1.398	6.335	0.171	0.000
4	3.852	26.856	1.099	3.788	0.050	0.820
5	0.208	17.944	4.715	12.047	0.991	0.905
6	0.781	7.271	1.526	5.566	0.391	0.119
7	-0.019	10.603	2.862	8.355	1.000	0.658
8	0.820	4.094	0.714	3.068	0.046	0.035
9	2.074	10.362	0.878	3.345	0.998	0.986
10	0.189	8.132	1.862	4.006	1.000	0.917
11	1.053	4.623	1.166	5.572	0.286	0.111
12	5.000	36.879	0.769	4.117	0.048	0.642
13	2.189	11.874	0.837	3.345	0.999	0.978
14	0.516	5.466	1.251	3.658	0.500	0.993
15	0.283	5.079	0.980	5.179	0.056	0.004
16	0.773	7.654	1.467	8.492	1.000	0.213
17	1.243	7.134	0.733	3.004	0.055	0.583
18	0.498	5.795	2.259	7.531	0.001	0.971
19	4.999	26.589	0.760	3.463	0.707	0.962
median	0.820	7.654	1.251	4.117	0.500	0.820
st.dev	1.524	8.830	0.940	2.320	0.410	0.388
20	-1.941	2.050	1.116	5.027	0.016	0.004
21	4.992	33.448	0.715	5.430	0.501	0.983
22	0.519	11.595	1.155	6.473	0.627	0.000
23	1.674	12.592	1.533	7.625	0.000	0.032
24	2.051	10.555	0.473	3.338	0.000	0.905
25	0.087	6.309	1.308	4.381	1.000	0.580
26	0.769	10.449	0.884	5.604	0.653	0.266
27	-1.393	16.408	1.086	9.218	0.137	0.038
28	1.316	10.976	0.180	5.249	1.000	0.368
median	0.769	10.976	1.086	5.430	0.501	0.266
st.dev	1.915	8.249	0.399	1.654	0.390	0.367
29	0.924	15.564	3.279	15.971	0.025	0.044
30	0.661	11.765	3.081	17.990	0.812	0.061
31	0.667	16.039	3.894	14.038	0.252	0.018
32	0.724	1.000	1.372	6.374	0.011	0.000
33	0.618	6.755	2.271	8.225	0.016	0.013
34	0.530	8.487	1.837	11.694	0.176	0.140
35	0.532	10.115	4.999	31.828	0.846	0.251
36	2.795	31.237	1.623	7.506	0.814	1.000
median	0.664	10.940	2.676	12.866	0.214	0.052
st.dev	0.714	8.370	1.171	7.716	0.361	0.316

Note. Parameters were constrained to lie within the following bounds: beta_V: -5-5; sigma_V: 1-1e2; beta_I: -5-5; sigma_I: 1-1e2; PrV: 0-1; PrC: 0-1.

Supplementary material Table S8. Biased response models negative log-likelihood scores (nLL). Lowest values are boldfaced.

	nLL					
pp	CC_V	CC_I	FF	SS	CI	n_obs
1	1534.61	1079.15	1078.04	1078.20	**1076.96**	400
2	1533.89	1175.79	1170.87	1175.79	**1170.86**	400
3	1548.08	1389.39	1388.96	**1368.30**	1368.30	400
4	1444.39	1105.29	1100.58	1105.29	**1100.57**	400
5	1848.45	1486.15	1485.57	1486.15	**1485.56**	400
6	1440.72	1288.42	1245.27	1288.42	**1240.03**	400
7	1614.03	1330.90	1330.89	1330.90	**1330.47**	399
8	1293.03	1056.19	1044.52	1029.83	**1016.24**	400
9	1328.87	1072.59	1034.11	1072.59	**1033.40**	400
10	1542.25	1083.11	1081.69	1083.11	**1081.68**	400
11	1266.30	1219.17	1149.07	1132.06	**1110.88**	375
12	1373.03	1156.83	1154.43	1151.86	**1150.02**	400
13	1319.61	1059.71	1032.45	1059.71	**1031.93**	398
14	1357.33	1037.91	1012.01	1037.91	**1012.01**	400
15	1437.22	1229.19	1229.16	1229.19	**1226.66**	400
16	1353.24	1337.22	1277.60	1296.83	**1274.95**	400
17	1272.56	1033.88	1001.65	1018.37	**989.69**	400
18	1383.40	1217.58	1177.91	1208.66	**1177.80**	400
19	1340.58	1101.11	1090.97	1101.11	**1086.73**	400
sum	27231.59	22459.59	22085.75	22254.29	**21964.76**	7572
20	1576.77	1247.50	1247.57	1238.83	**1238.73**	400
21	1442.45	1251.83	1240.76	1251.83	**1240.66**	400
22	1562.66	1542.47	1526.81	1526.72	**1526.37**	399
23	1662.51	1371.99	1368.61	1371.99	**1362.46**	399
24	1281.46	1082.84	1035.88	1082.84	**1035.35**	400
25	1442.42	1148.26	1138.88	1147.16	**1135.70**	400
26	1407.66	1269.31	1241.60	1245.87	**1230.55**	399
27	1602.78	1453.13	1454.11	1451.27	**1451.26**	394
28	1240.52	1341.34	1219.02	1223.34	**1215.79**	400
sum	13219.23	11708.66	11473.23	11539.85	**11436.88**	3591
29	2470.53	2232.92	2193.99	2232.92	**2176.35**	525
30	2141.72	2373.67	2091.33	2091.58	**2072.27**	525
31	2485.01	2319.81	2259.24	2318.00	**2233.99**	525
32	2296.20	1754.18	1754.70	1740.22	**1731.46**	525
33	2366.66	1900.51	1895.13	1879.61	**1867.05**	525
34	2161.73	2104.68	1958.55	1997.18	**1925.37**	525
35	1992.39	2314.09	1963.47	1984.49	**1961.95**	525
36	2219.30	1928.20	1816.49	1928.20	**1806.30**	525
sum	18133.53	16928.05	15932.92	16172.21	**15774.74**	4200
overall	58584.35	51096.30	49491.90	49966.35	**49176.39**	15363.00

Supplementary material Table S9. Biased response models Bayesian Information Criterion scores (BIC). Best model scores are boldfaced.

	BIC				
pp	CC_V	CC_I	FF	SS	CI
1	3077.53	**2166.61**	2168.55	2173.02	2174.69
2	3076.08	2359.88	**2354.21**	2368.19	2362.49
3	3104.47	2787.09	2790.38	**2753.22**	2757.37
4	2897.09	2218.88	**2213.62**	2227.19	2221.90
5	3705.20	**2980.62**	2983.60	2988.92	2991.89
6	2889.75	2585.15	2503.00	2593.46	**2500.83**
7	3236.36	**2670.09**	2674.23	2678.40	2681.70
8	2594.37	2120.68	2101.50	2076.28	**2053.25**
9	2666.04	2153.48	**2080.68**	2161.79	2087.57
10	3092.81	**2174.53**	2175.84	2182.84	2184.13
11	2540.79	2446.51	2310.40	2280.47	**2242.21**
12	2754.36	2321.97	2321.32	**2320.34**	2320.81
13	2647.51	2127.72	**2077.35**	2136.02	2084.60
14	2722.96	2084.14	**2036.48**	2092.44	2044.79
15	2882.74	**2466.70**	2470.79	2475.00	2474.09
16	2714.79	2682.76	**2567.66**	2610.28	2570.67
17	2553.42	2076.06	2015.76	2053.35	**2000.14**
18	2775.11	2443.47	**2368.28**	2433.93	2376.38
19	2689.46	2210.52	2194.40	2218.83	**2194.23**
iteration 1	54732.76	45188.76	**44575.88**	45047.75	44603.48
20	3161.86	2503.31	2507.61	**2494.28**	2498.24
21	2893.20	2511.97	**2493.97**	2520.27	2502.10
22	3133.62	3093.23	**3066.08**	3070.05	3073.50
23	3333.32	2752.28	2749.66	2760.58	**2745.67**
24	2571.23	2173.99	**2084.22**	2182.29	2091.48
25	2893.15	2304.82	**2290.21**	2310.93	2292.17
26	2823.62	2546.91	2495.65	2508.35	**2481.85**
27	3213.84	**2914.55**	2920.64	2919.09	2923.21
28	2489.34	2690.99	**2450.49**	2463.30	2452.35
iteration 2	26552.73	23531.59	**23117.86**	23308.24	23159.43
29	4949.90	4474.68	4401.25	4483.54	**4374.82**
30	4292.29	4756.20	4195.94	4200.86	**4166.68**
31	4978.86	4648.47	4531.77	4653.69	**4490.11**
32	4601.25	3517.21	3522.67	3498.15	**3485.05**
33	4742.18	3809.87	3803.54	3776.93	**3756.24**
34	4332.31	4218.21	3930.39	4012.07	**3872.87**
35	3993.62	4637.03	**3940.22**	3986.69	3946.02
36	4447.46	3865.25	3646.26	3874.10	**3634.72**
iteration 3	36371.14	33960.18	32021.96	32552.58	**31809.68**

Supplementary material Table S10. Biased response Visual Cue Capture model parameter estimates.

pp	beta_R	sigma_V
1	0.500	23.131
2	0.500	23.071
3	0.500	23.964
4	0.500	18.277
5	0.500	56.696
6	0.500	18.049
7	0.500	28.905
8	0.500	12.406
9	0.500	13.576
10	0.500	23.600
11	0.523	13.662
12	0.500	15.217
13	0.500	13.490
14	0.500	14.605
15	0.500	17.955
16	0.502	14.348
17	0.500	11.771
18	0.500	15.589
19	0.500	14.000
median	0.500	15.589
st.dev	0.005	9.921
20	0.500	25.974
21	0.500	18.220
22	0.500	25.112
23	0.500	33.070
24	0.500	12.068
25	0.500	18.231
26	0.500	16.784
27	0.500	29.876
28	0.500	10.847
median	0.500	18.231
st.dev	0.111	6.284
29	0.500	67.206
30	0.548	27.030
31	0.500	67.918
32	0.500	47.123
33	0.500	54.584
34	0.500	32.933
35	0.505	21.802
36	0.500	38.037
median	0.500	42.580
st.dev	0.088	17.411

Note. Parameters were constrained to lie within the following bounds: beta_R: 0.5-5; sigma_V: 1-1e2; sigma_I: 1-1e2; PrV: 0-1; PrC: 0-1.

Supplementary material Table S11. Biased response Inertial Cue Capture model parameter estimates.

pp	beta_R	sigma_I
1	1.440	2.497
2	1.401	3.266
3	1.145	6.829
4	1.076	3.567
5	3.170	3.137
6	0.989	6.143
7	1.643	4.141
8	0.628	5.410
9	0.785	4.505
10	1.473	2.463
11	0.703	8.918
12	0.756	5.777
13	0.767	4.528
14	0.864	3.755
15	0.883	5.930
16	0.574	12.019
17	0.636	5.050
18	0.855	5.953
19	0.738	5.146
median	0.864	5.050
st.dev	0.582	2.236
20	1.118	4.902
21	0.695	7.987
22	0.546	21.650
23	1.452	5.196
24	0.500	7.278
25	0.768	5.572
26	0.627	9.329
27	0.912	10.663
28	0.500	14.065
median	0.695	7.987
st.dev	0.303	5.077
29	2.940	5.797
30	0.900	25.503
31	2.936	6.856
32	1.374	4.983
33	2.202	4.106
34	1.158	11.583
35	0.562	37.797
36	1.529	6.240
median	1.452	6.548
st.dev	0.843	11.466

Note. Parameters were constrained to lie within the following bounds: beta_R: 0.5-5; sigma_V: 1-1e2; sigma_I: 1-1e2; PrV: 0-1; PrC: 0-1.

Supplementary material Table S12. Biased response Switching Strategy model parameter estimates.

pp	beta_R	sigma_V	sigma_I	PrV
1	1.441	5.795	2.432	0.006
2	1.401	23.542	3.266	0.000
3	1.289	4.369	5.151	0.114
4	1.076	22.850	3.567	0.000
5	3.170	22.953	3.137	0.000
6	0.989	22.709	6.143	0.000
7	1.643	23.551	4.141	0.000
8	0.675	4.829	4.166	0.108
9	0.785	22.102	4.505	0.000
10	1.473	22.998	2.463	0.000
11	0.939	3.651	4.561	0.473
12	0.766	12.574	5.428	0.016
13	0.767	22.383	4.528	0.000
14	0.864	22.422	3.755	0.000
15	0.883	22.326	5.930	0.000
16	0.811	6.989	6.384	0.410
17	0.659	3.746	4.376	0.080
18	0.891	5.126	5.116	0.103
19	0.738	22.544	5.146	0.000
median	0.891	22.326	4.505	0.000
st.dev	0.564	8.525	1.119	0.134
20	1.130	18.165	4.600	0.008
21	0.695	22.687	7.987	0.000
22	0.817	13.971	11.298	0.416
23	1.452	23.216	5.196	0.000
24	0.500	23.085	7.278	0.000
25	0.776	6.591	5.356	0.013
26	0.706	3.832	7.294	0.109
27	0.943	24.236	9.761	0.023
28	0.500	9.694	8.406	0.754
median	0.776	18.165	7.294	0.013
st.dev	0.287	7.452	2.081	0.249
29	2.940	25.354	5.797	0.000
30	0.647	14.995	23.523	0.770
31	3.018	29.170	6.312	0.013
32	1.368	25.504	4.708	0.006
33	2.226	31.539	3.830	0.003
34	0.500	13.562	22.970	0.556
35	0.509	19.060	49.841	0.950
36	1.529	25.664	6.240	0.000
median	1.448	25.429	6.276	0.009
st.dev	0.972	6.108	15.068	0.378

Note. Parameters were constrained to lie within the following bounds: beta_R: 0.5-5; sigma_V: 1-1e2; sigma_I: 1-1e2; PrV: 0-1; PrC: 0-1.

Supplementary material Table S13. Biased response Forced Fusion model parameter estimates.

pp	beta_R	sigma_V	sigma_I
1	1.477	15.168	2.458
2	1.502	11.619	3.118
3	1.196	31.516	6.688
4	1.159	12.250	3.399
5	3.246	19.985	3.097
6	1.365	7.586	4.697
7	1.649	67.424	4.137
8	0.742	11.332	4.840
9	0.994	7.046	3.639
10	1.517	14.137	2.420
11	1.176	6.818	5.791
12	0.824	18.440	5.511
13	0.941	7.975	3.822
14	1.023	7.517	3.235
15	0.892	57.436	5.909
16	1.061	8.182	7.628
17	0.811	7.863	4.130
18	1.158	7.749	4.635
19	0.857	11.580	4.661
median	1.158	11.580	4.137
st.dev	0.539	16.590	1.376
20	1.121	99.979	4.902
21	0.834	15.819	7.107
22	0.880	20.320	16.534
23	1.558	18.392	4.980
24	0.613	9.976	6.221
25	0.867	14.226	5.128
26	0.853	12.350	7.477
27	0.925	100.000	10.628
28	0.500	12.041	19.921
median	0.867	15.819	7.107
st.dev	0.285	35.573	5.162
29	3.117	21.199	5.235
30	1.369	15.612	12.136
31	3.190	19.763	5.870
32	1.378	99.997	4.979
33	2.239	31.364	4.034
34	1.397	17.249	8.007
35	0.985	14.863	14.722
36	1.685	14.907	4.810
median	1.541	18.506	5.553
st.dev	0.786	27.173	3.662

Note. Parameters were constrained to lie within the following bounds: beta_R: 0.5-5; sigma_V: 1-1e2; sigma_I: 1-1e2; PrV: 0-1; PrC: 0-1.

Supplementary material Table S14. Biased response Causal Inference model parameter estimates.

pp	beta_R	sigma_V	sigma_I	PrV	PrC
1	1.460	16.760	2.377	0.018	0.156
2	1.502	11.701	3.115	0.500	0.976
3	1.289	4.372	5.157	0.114	0.000
4	1.159	12.404	3.395	0.500	0.952
5	3.246	19.022	3.100	0.000	0.474
6	1.311	8.410	4.363	0.594	0.371
7	1.676	10.408	4.123	0.000	0.012
8	0.730	5.425	4.194	0.058	0.030
9	0.994	7.202	3.578	0.998	0.937
10	1.517	14.429	2.418	0.500	0.976
11	1.111	4.322	5.042	0.234	0.119
12	0.805	15.752	5.303	0.031	0.037
13	0.941	8.231	3.769	0.999	0.900
14	1.023	7.517	3.235	0.500	0.999
15	0.911	5.145	5.886	0.000	0.004
16	1.055	8.629	6.922	0.987	0.252
17	0.804	7.237	3.932	0.073	0.497
18	1.158	7.622	4.650	0.001	0.795
19	0.864	14.452	4.132	0.990	0.409
median	1.111	8.410	4.123	0.234	0.409
st.dev	0.541	4.302	1.133	0.379	0.387
20	1.136	19.533	4.586	0.009	0.009
21	0.834	16.691	7.000	0.926	0.664
22	0.878	21.374	15.018	0.616	0.072
23	1.543	8.087	4.943	0.000	0.034
24	0.613	9.568	6.237	0.000	0.647
25	0.877	17.344	4.732	1.000	0.572
26	0.855	12.996	6.462	0.637	0.282
27	0.943	25.079	9.807	0.023	0.000
28	0.500	12.874	13.966	1.000	0.038
median	0.877	16.691	6.462	0.616	0.072
st.dev	0.283	5.258	3.745	0.431	0.274
29	2.953	15.214	4.933	0.000	0.864
30	1.394	14.416	10.565	1.000	0.973
31	3.232	18.689	5.081	0.998	0.989
32	1.381	78.060	3.969	0.174	0.612
33	2.226	47.952	3.275	0.226	0.734
34	1.262	14.411	7.849	0.000	0.946
35	1.006	14.711	13.948	1.000	0.954
36	1.691	14.596	4.528	0.666	1.000
median	1.543	14.963	5.007	0.446	0.950
st.dev	0.772	22.009	3.507	0.427	0.131

Note. Parameters were constrained to lie within the following bounds: beta_R: 0.5-5; sigma_V: 1-1e2; sigma_I: 1-1e2; PrV: 0-1; PrC: 0-1.

Supplementary material Figure S1. Data and models fits for participant 1 (It. 1)

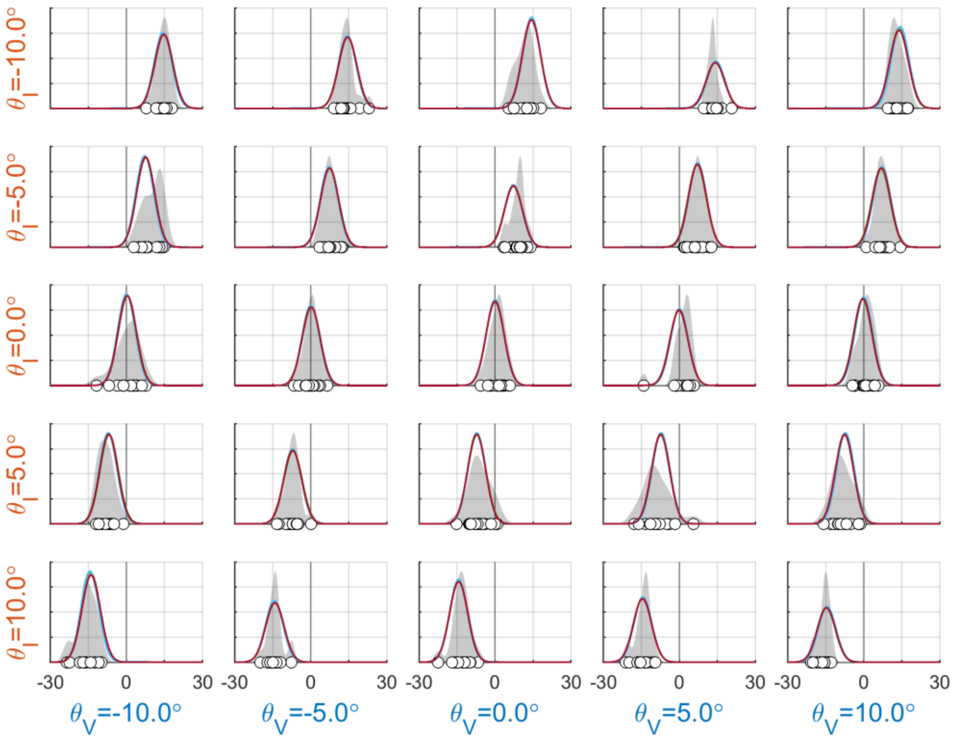

Supplementary material Figure S2. Data and models fits for participant 2 (It. 1)

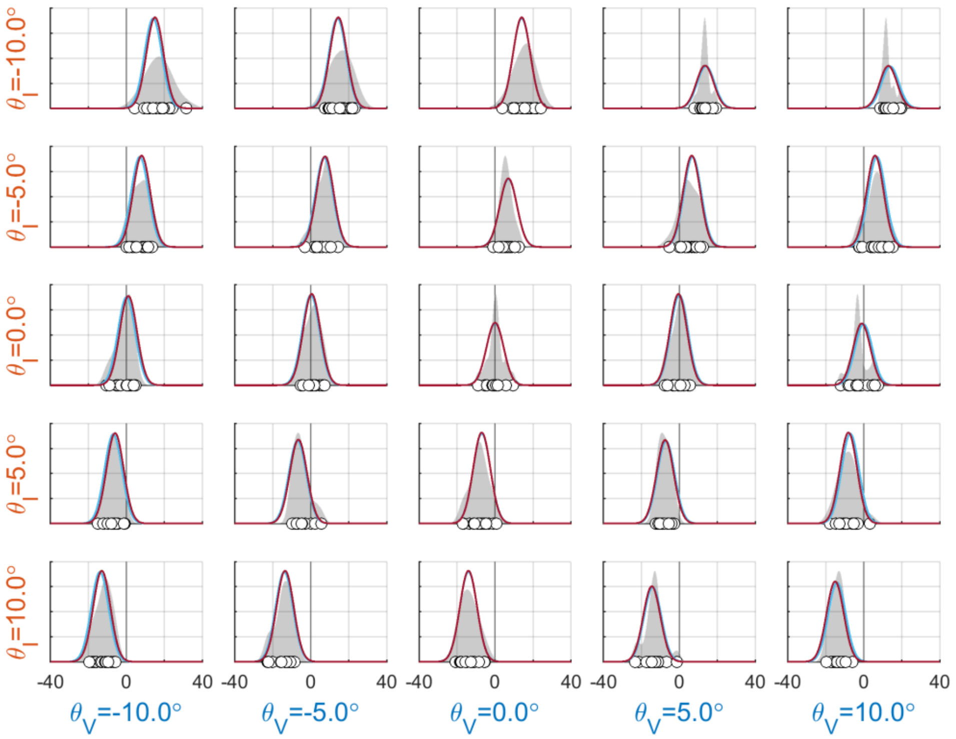

Supplementary material Figure S3. Data and models fits for participant 3 (It. 1)

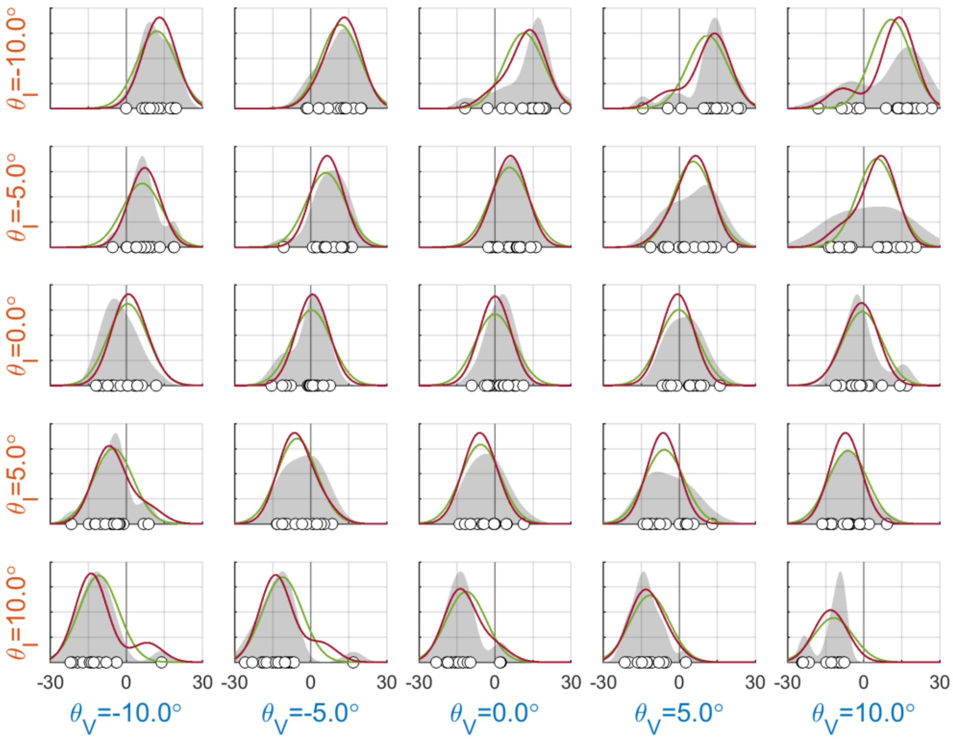

Supplementary material Figure S4. Data and models fits for participant 4 (It. 1)

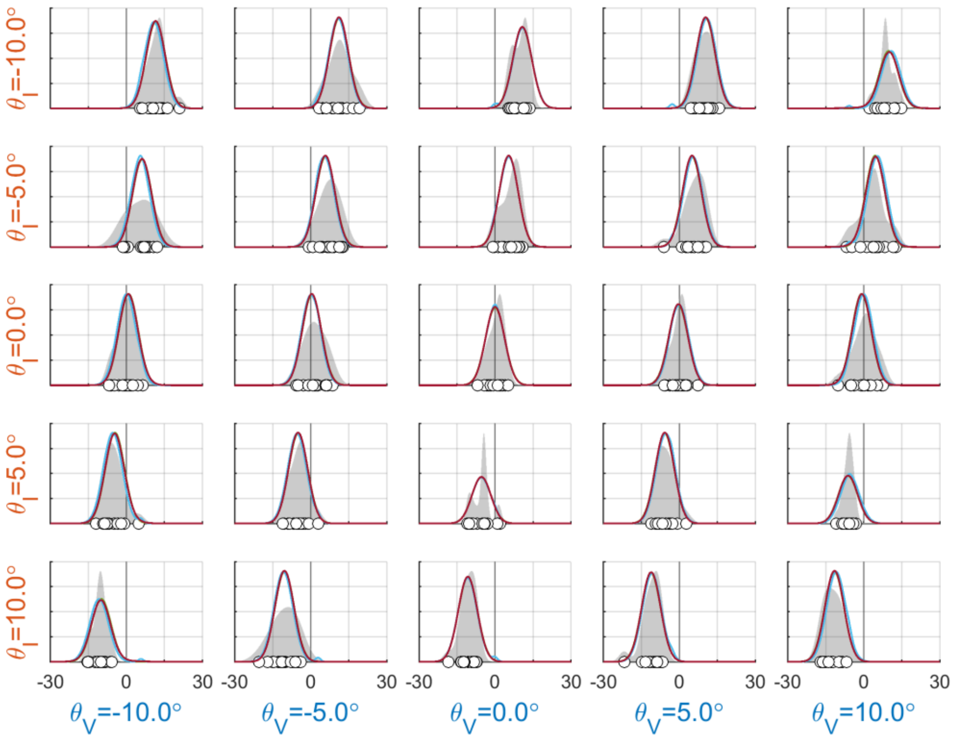

Supplementary material Figure S5. Data and models fits for participant 5 (It. 1)

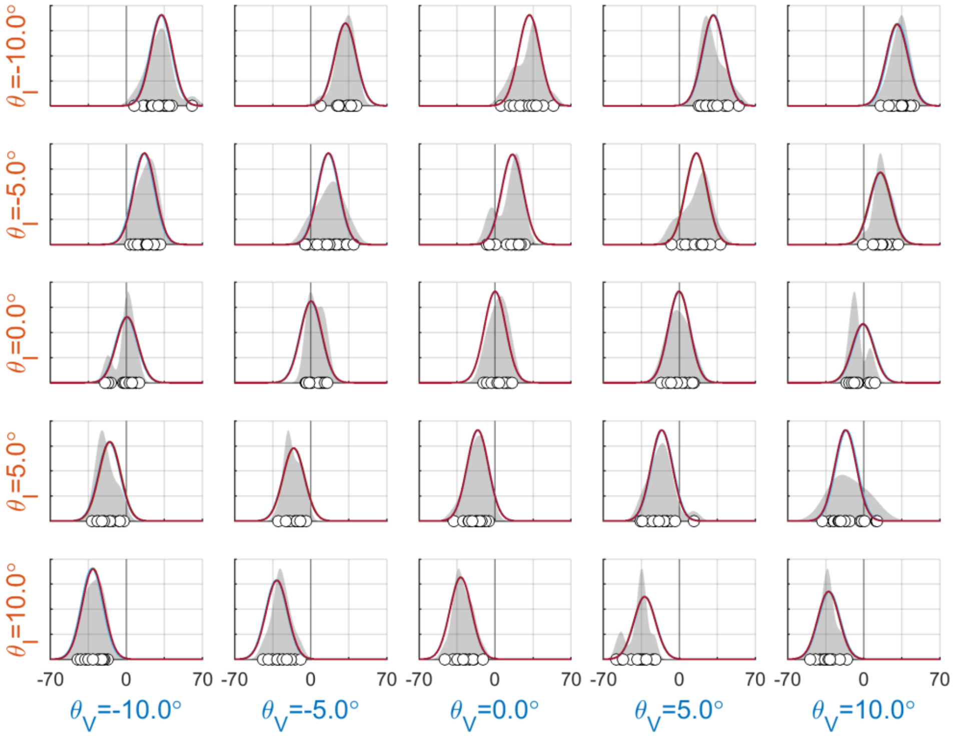

Supplementary material Figure S6. Data and models fits for participant 6 (It. 1)

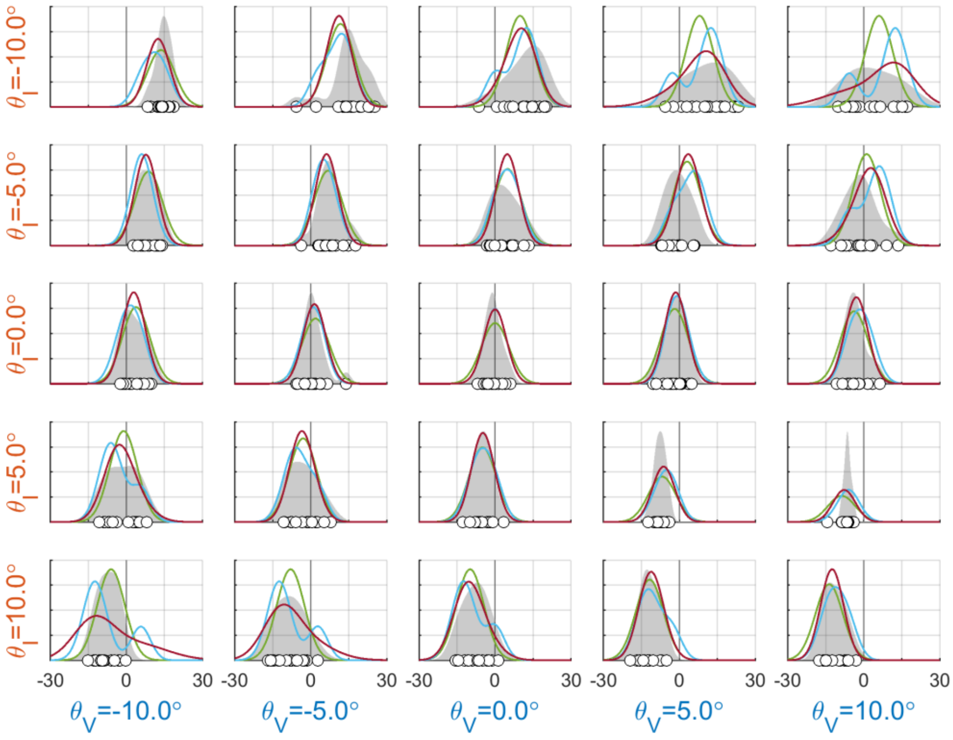

Supplementary material Figure S7. Data and models fits for participant 7 (It. 1)

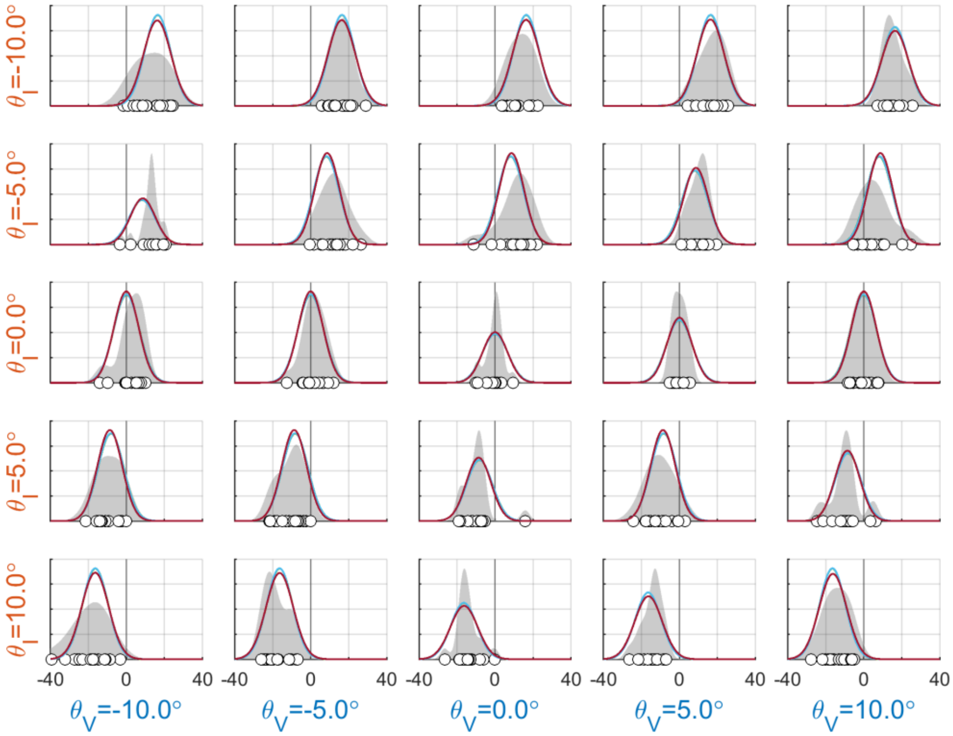

Supplementary material Figure S8. Data and models fits for participant 8 (It. 1)

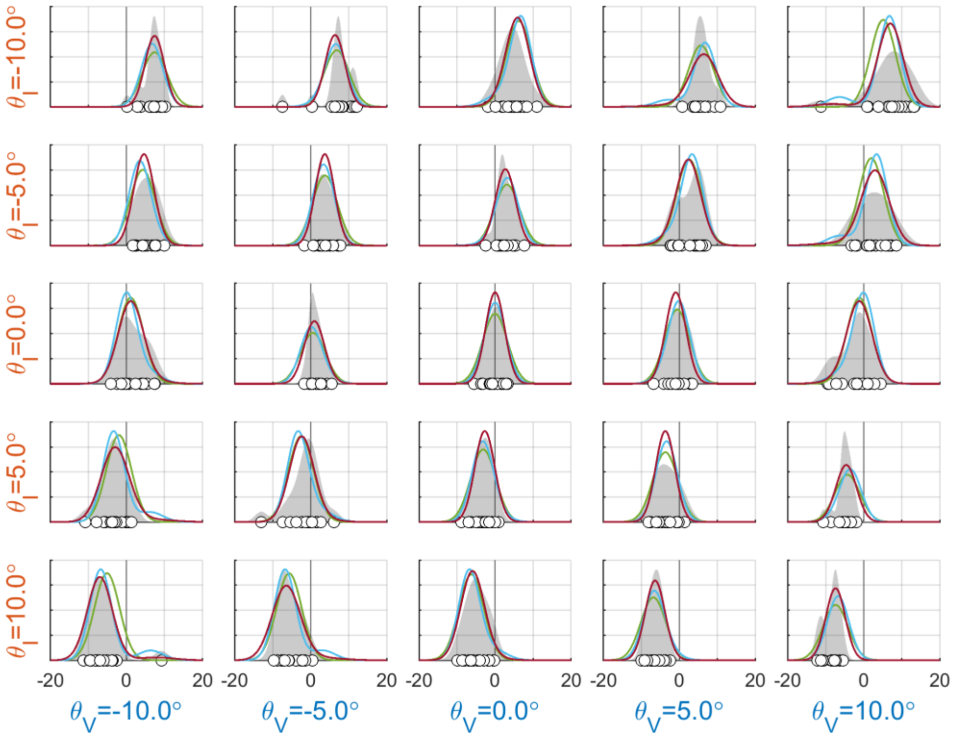

Supplementary material Figure S9. Data and models fits for participant 9 (It. 1)

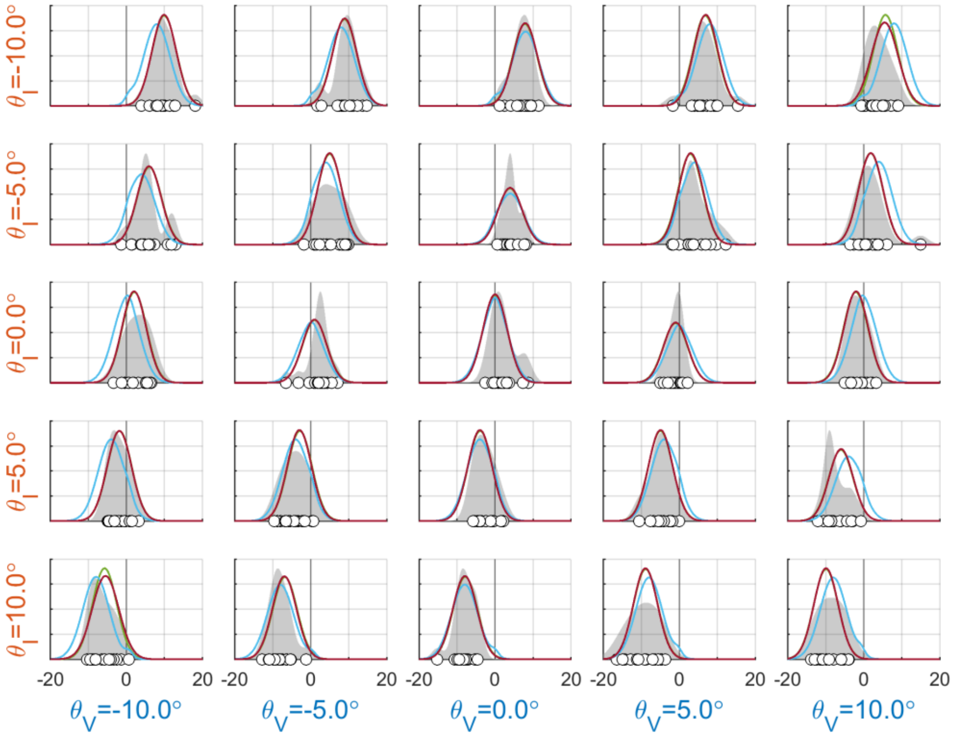

Supplementary material Figure S10. Data and models fits for participant 10 (It. 1)

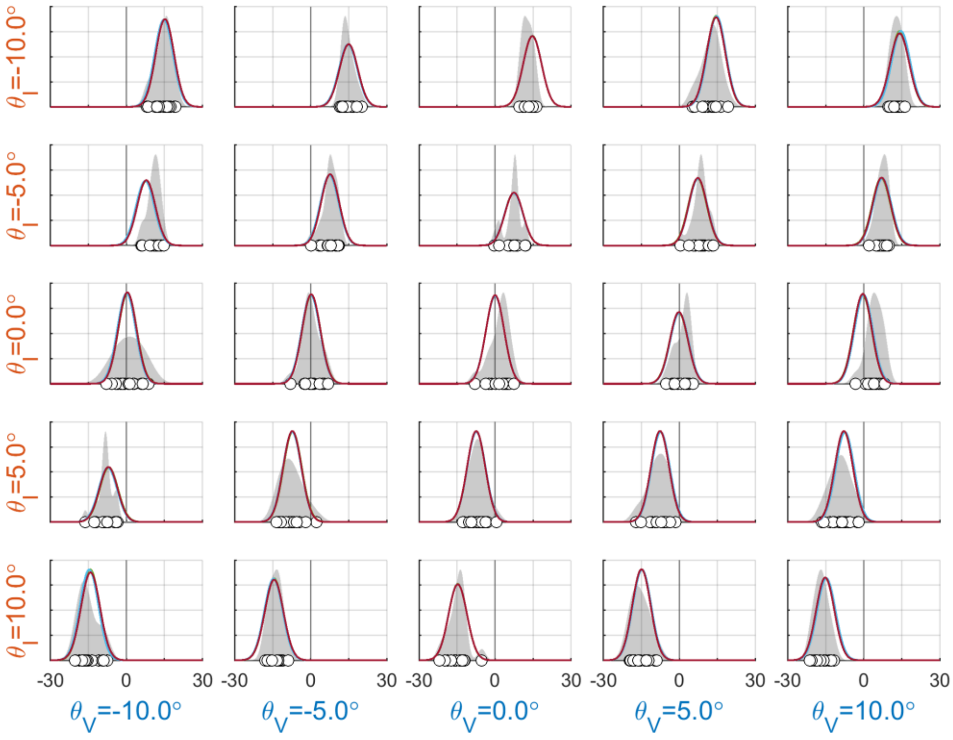

Supplementary material Figure S11. Data and models fits for participant 11 (It. 1)

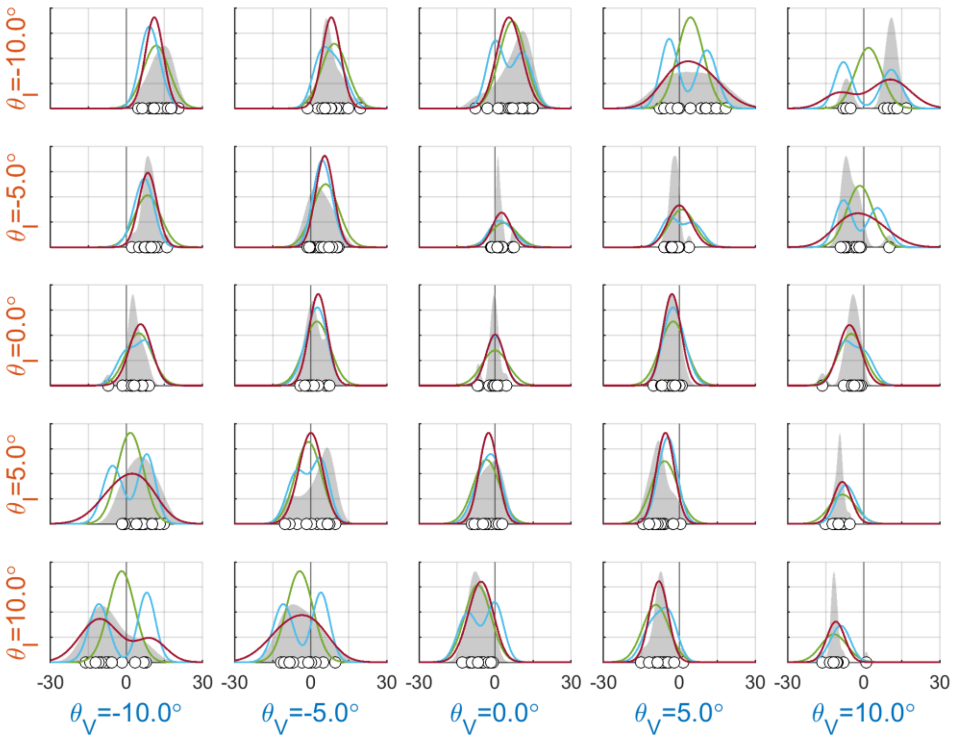

Supplementary material Figure S12. Data and models fits for participant 12 (It. 1)

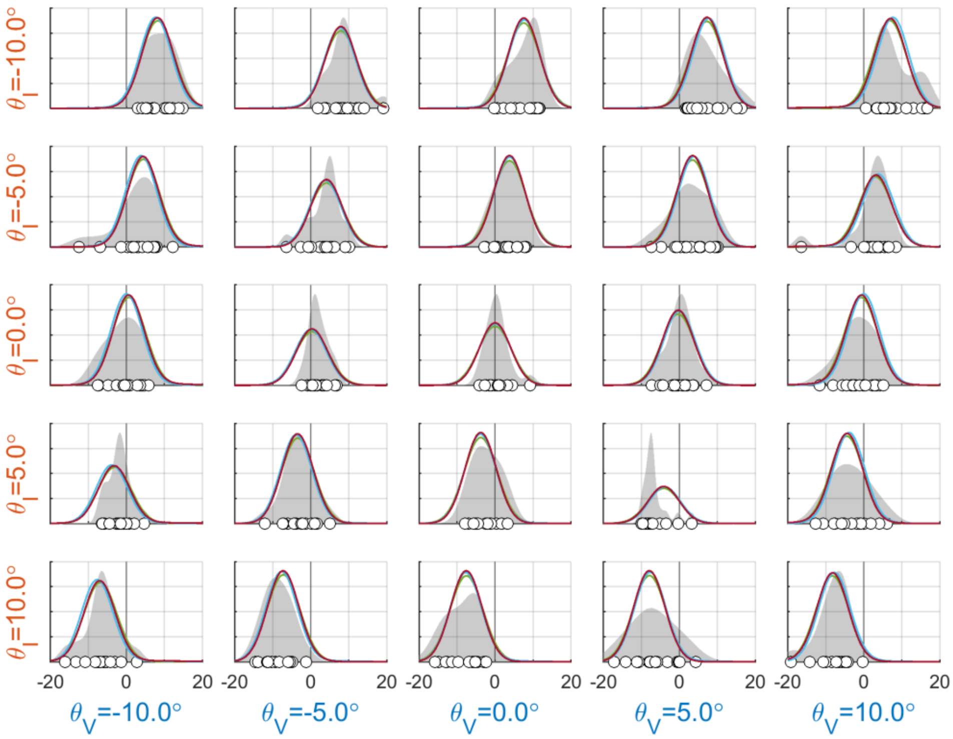

Supplementary material Figure S13. Data and models fits for participant 13 (It. 1)

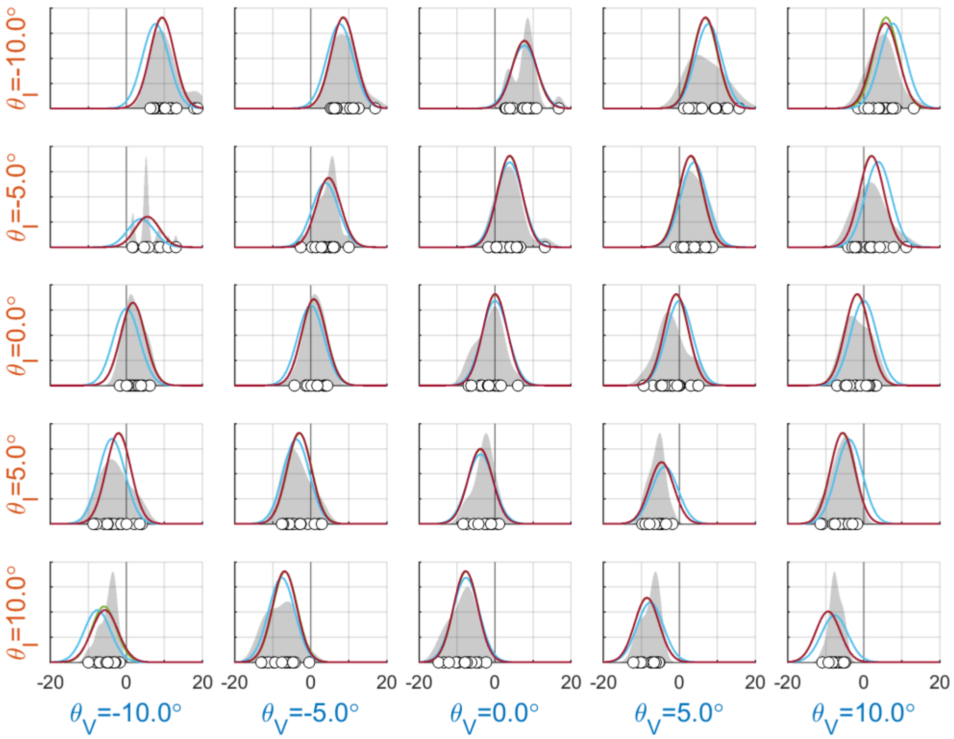

Supplementary material Figure S14. Data and models fits for participant 14 (It. 1)

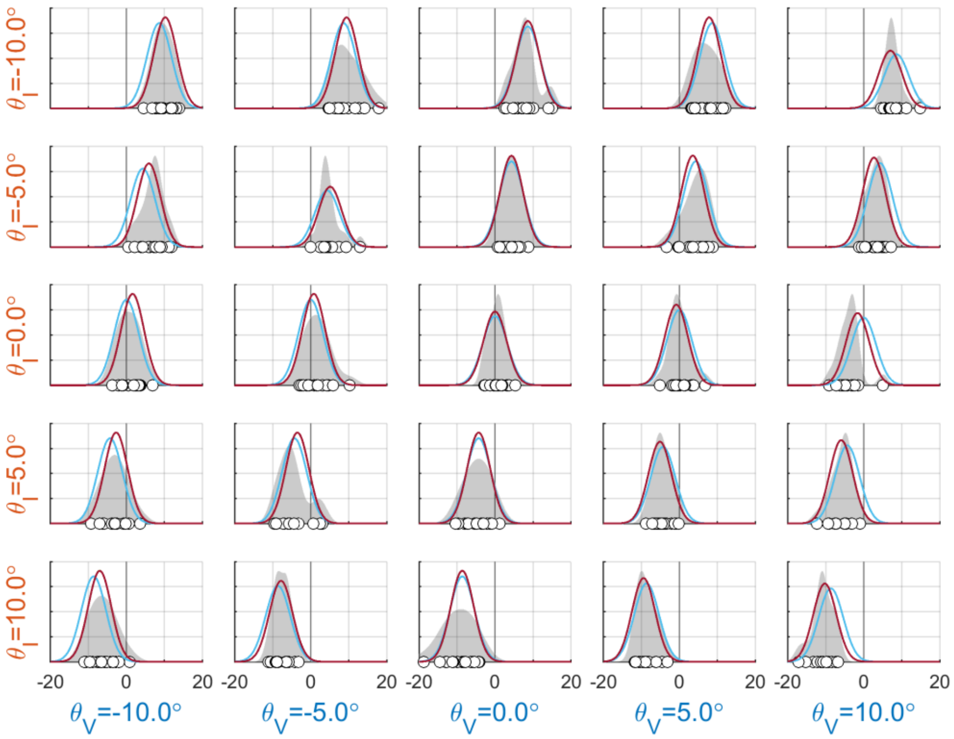

Supplementary material Figure S15. Data and models fits for participant 15 (It. 1)

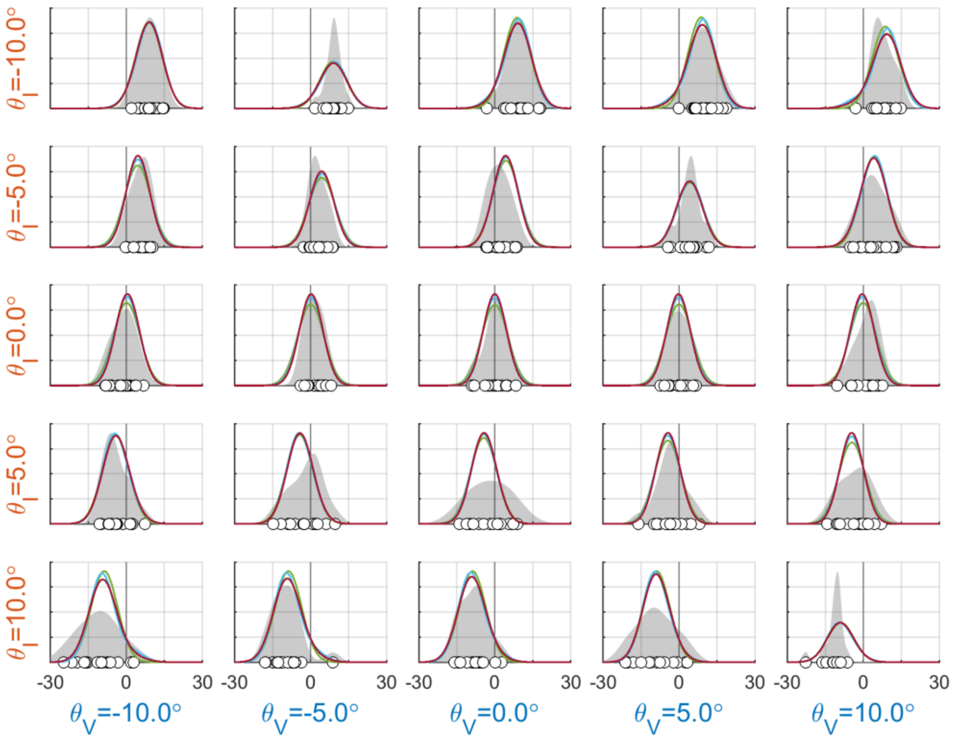

Supplementary material Figure S16. Data and models fits for participant 16 (It. 1)

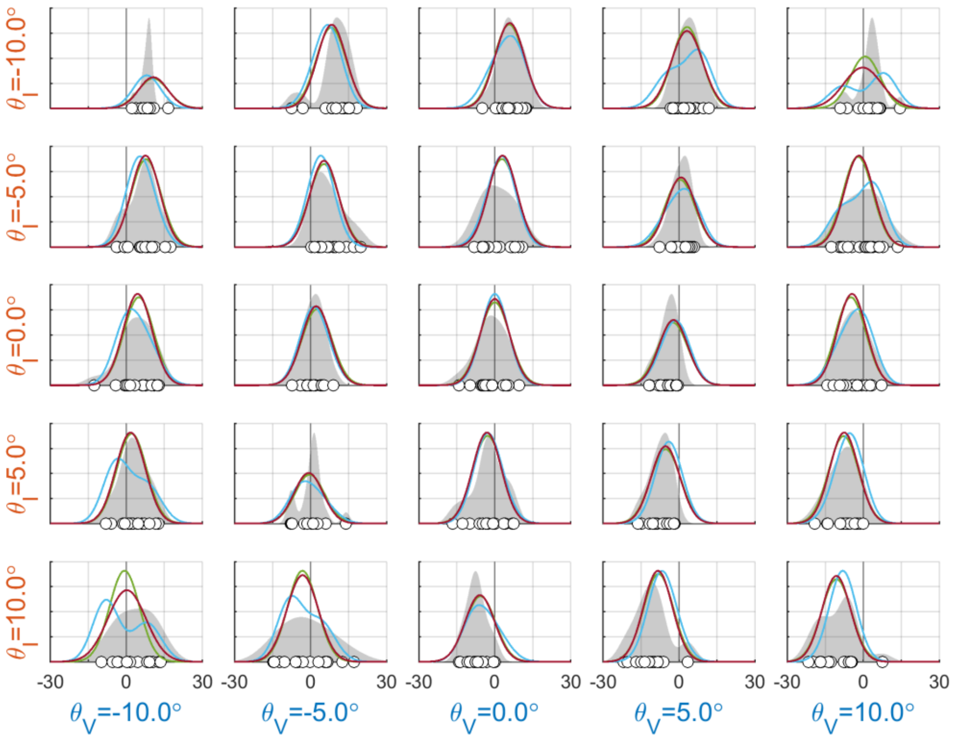

Supplementary material Figure S17. Data and models fits for participant 17 (It. 1)

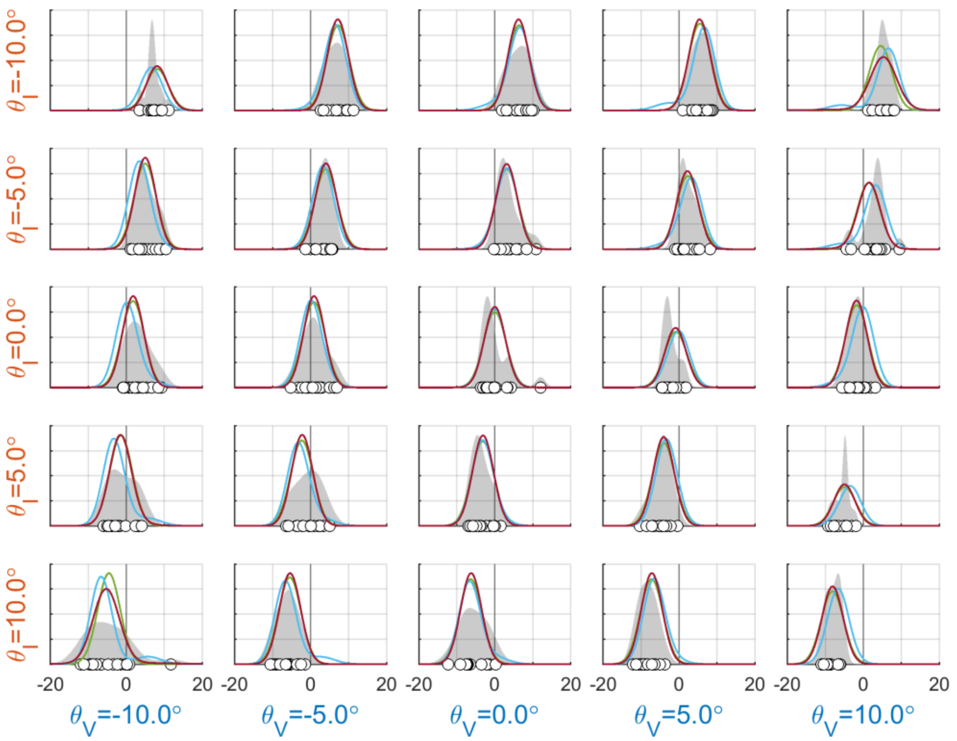

Supplementary material Figure S18. Data and models fits for participant 18 (It. 1)

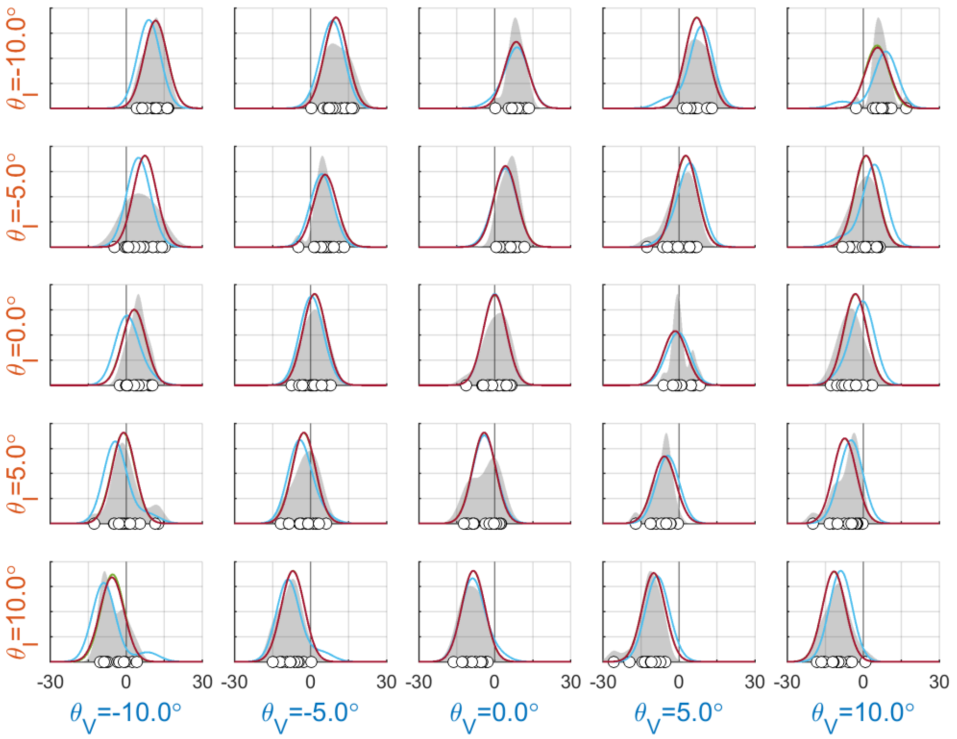

Supplementary material Figure S19. Data and models fits for participant 19 (It. 1)

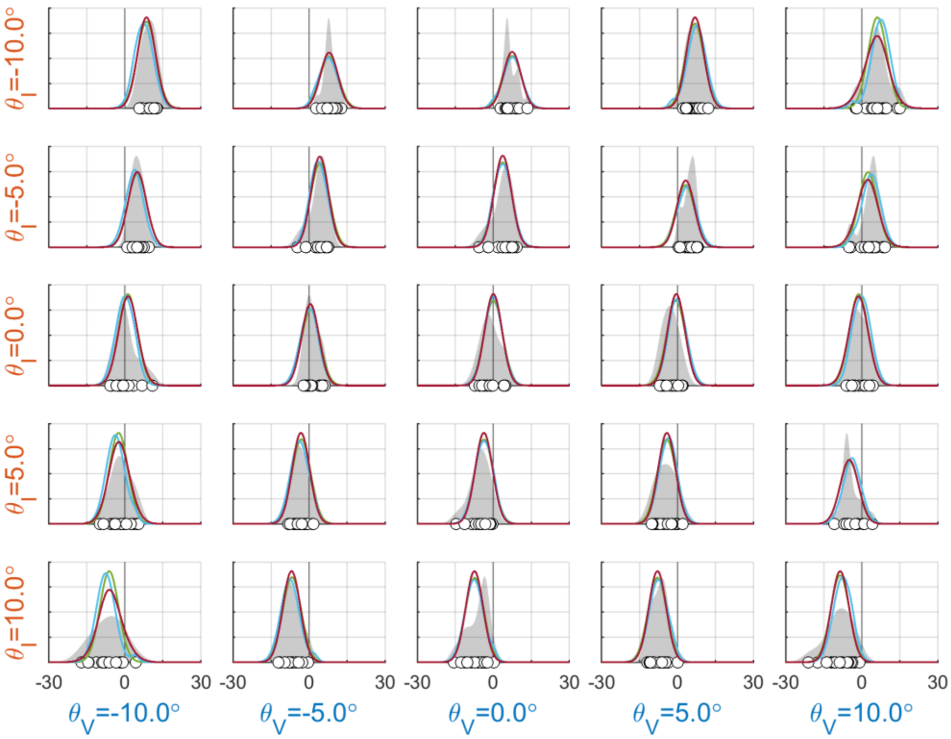

Supplementary material Figure S20. Data and models fits for participant 20 (It. 2)

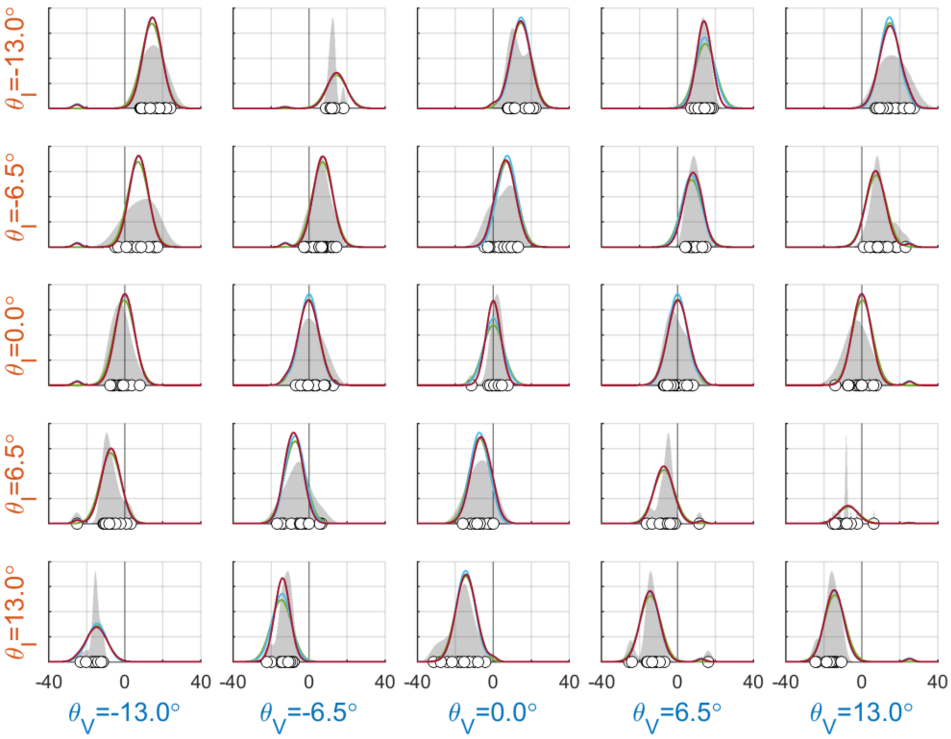

Supplementary material Figure S21. Data and models fits for participant 21 (It. 2)

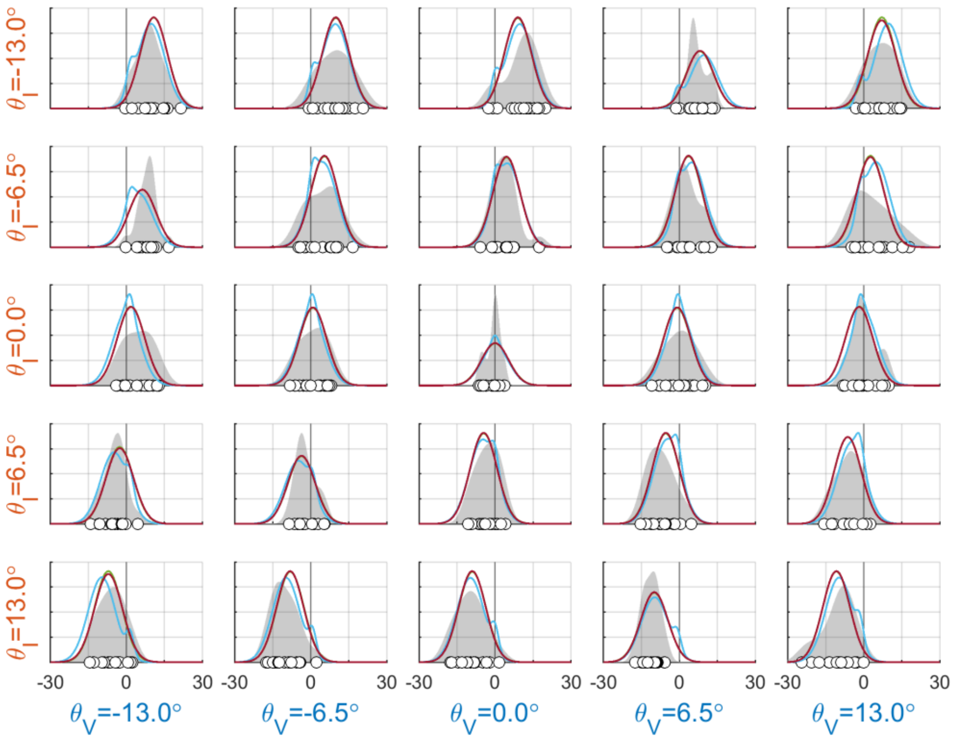

Supplementary material Figure S22. Data and models fits for participant 22 (It. 2)

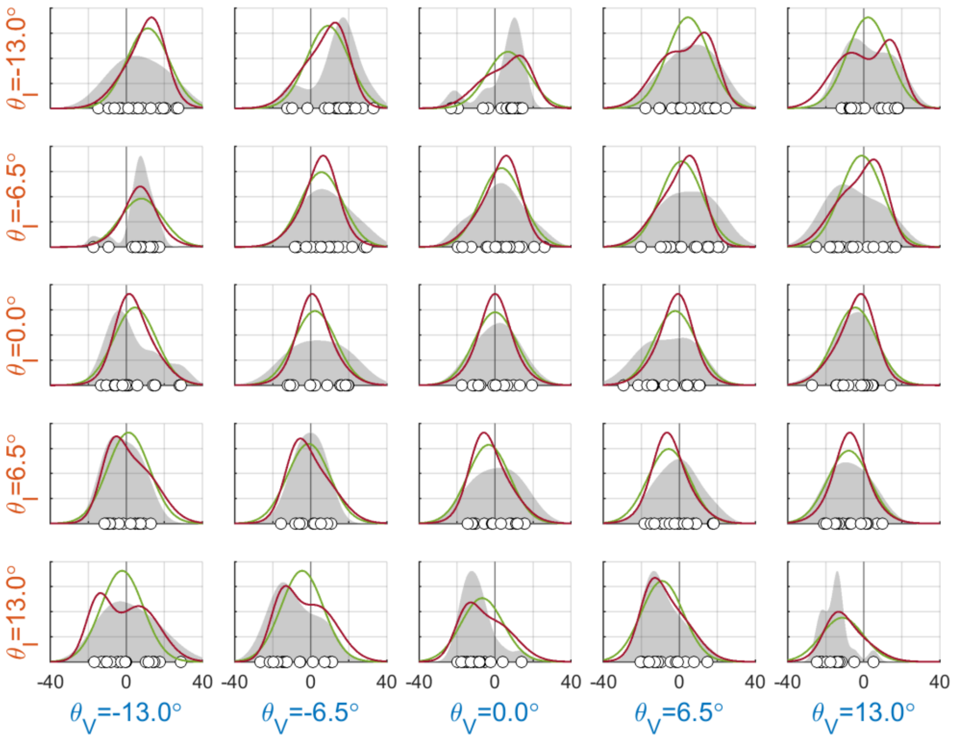

Supplementary material Figure S23. Data and models fits for participant 23 (It. 2)

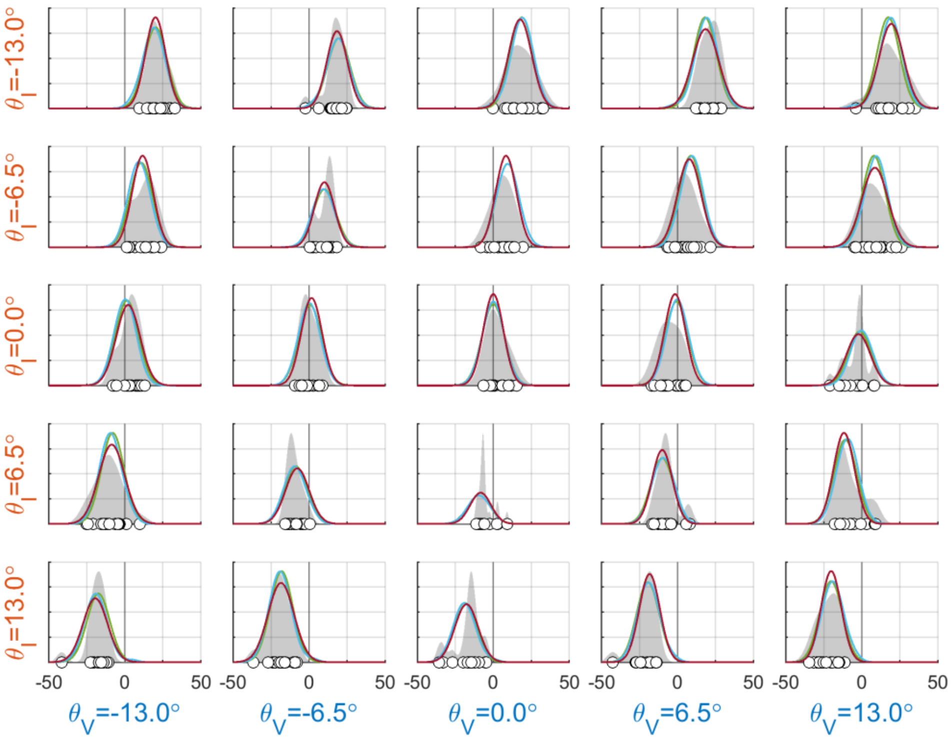

Supplementary material Figure S24. Data and models fits for participant 24 (It. 2)

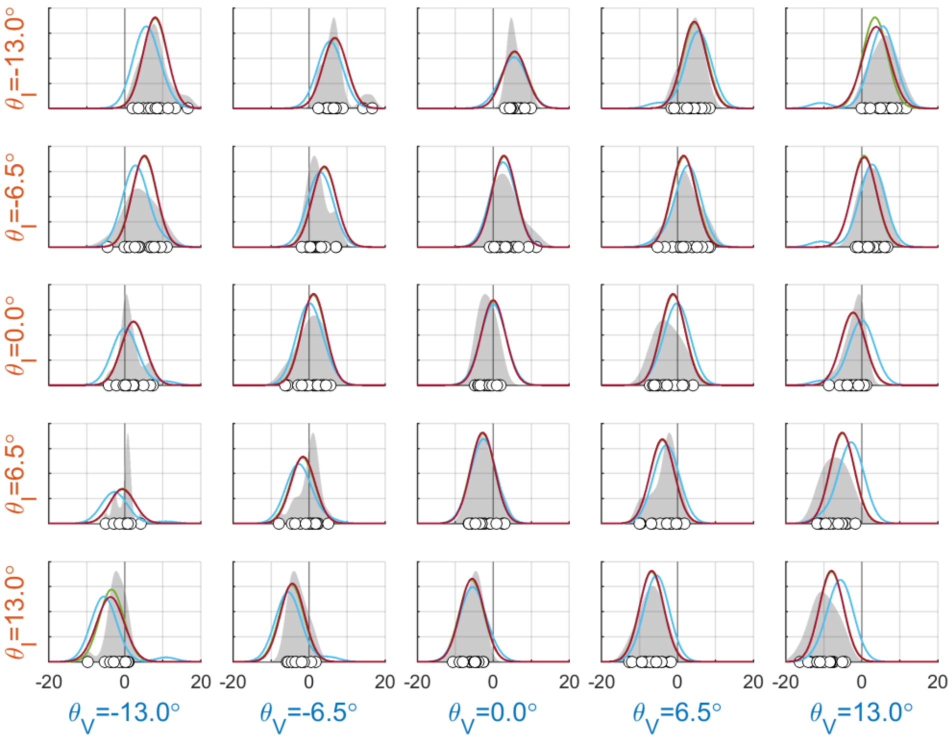

Supplementary material Figure S25. Data and models fits for participant 25 (It. 2)

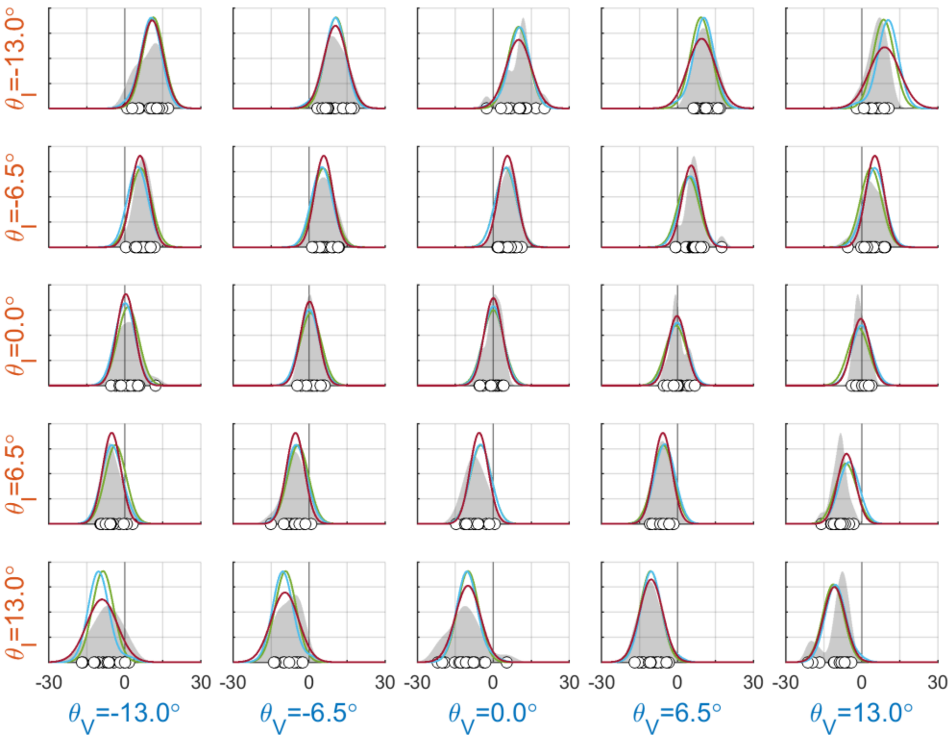

Supplementary material Figure S26. Data and models fits for participant 26 (It. 2)

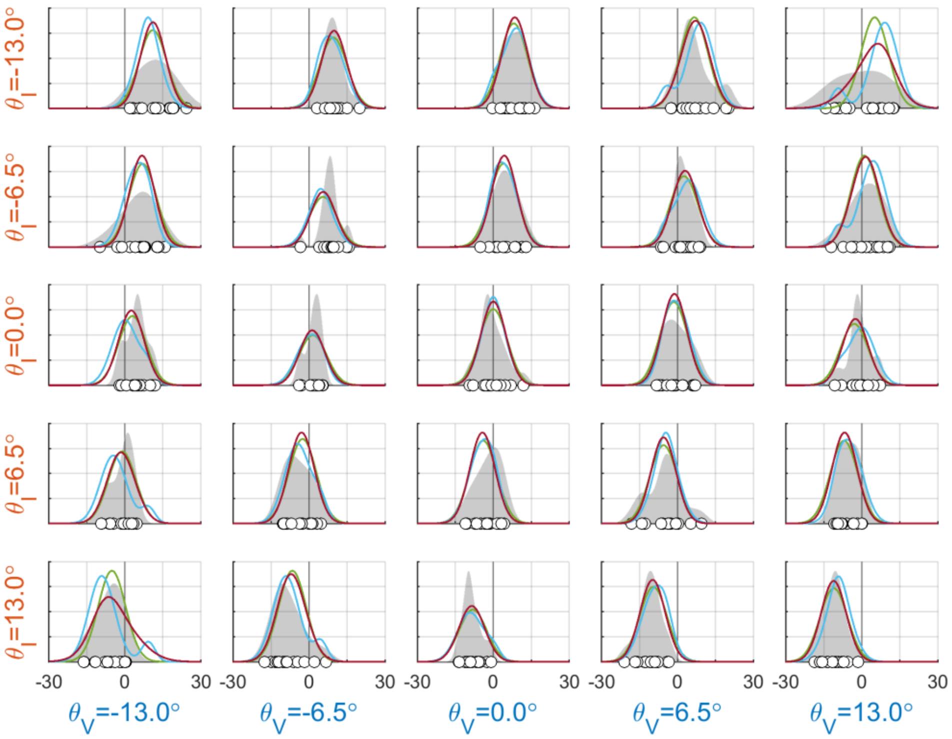

Supplementary material Figure S27. Data and models fits for participant 27 (It. 2)

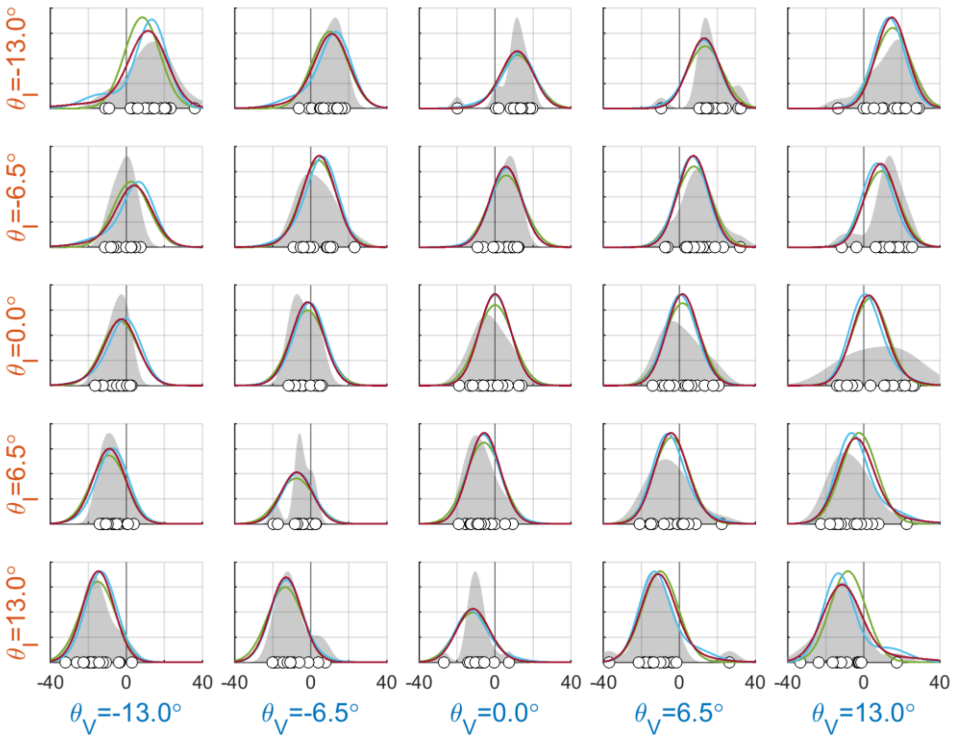

Supplementary material Figure S28. Data and models fits for participant 28 (It. 2)

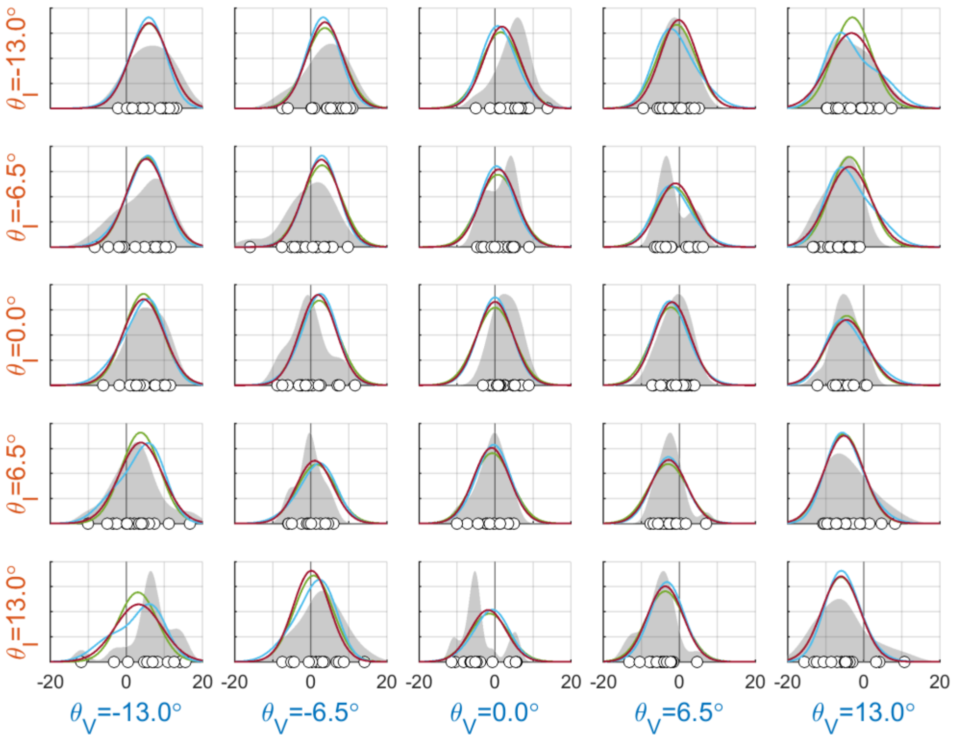

Supplementary material Figure S29. Data and models fits for participant 29 (It. 3)

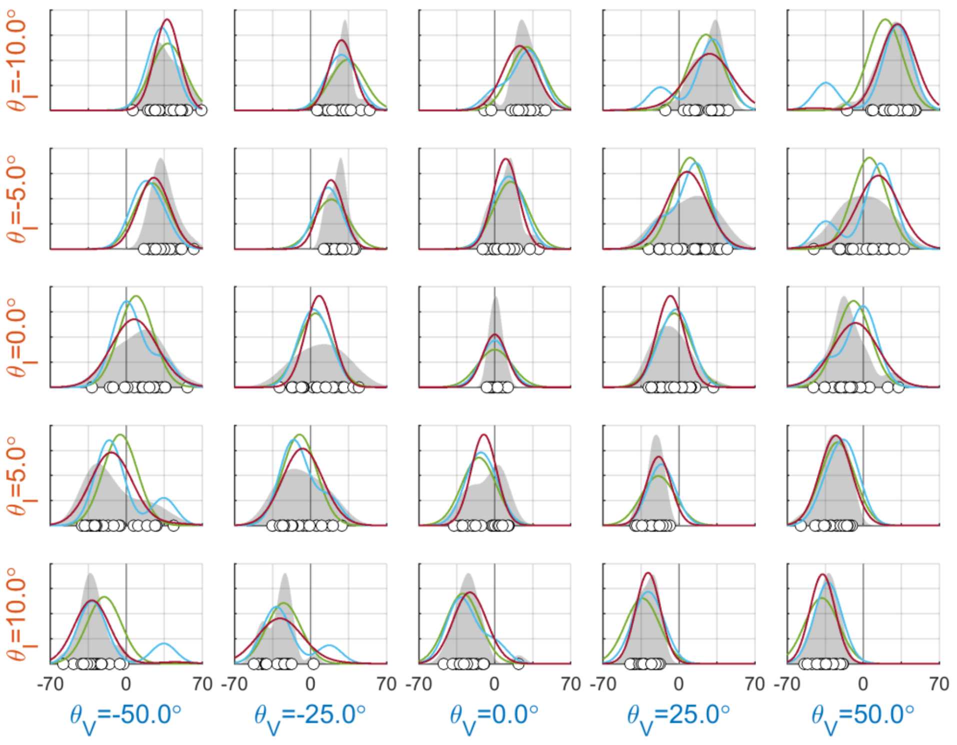

Supplementary material Figure S30. Data and models fits for participant 30 (It. 3)

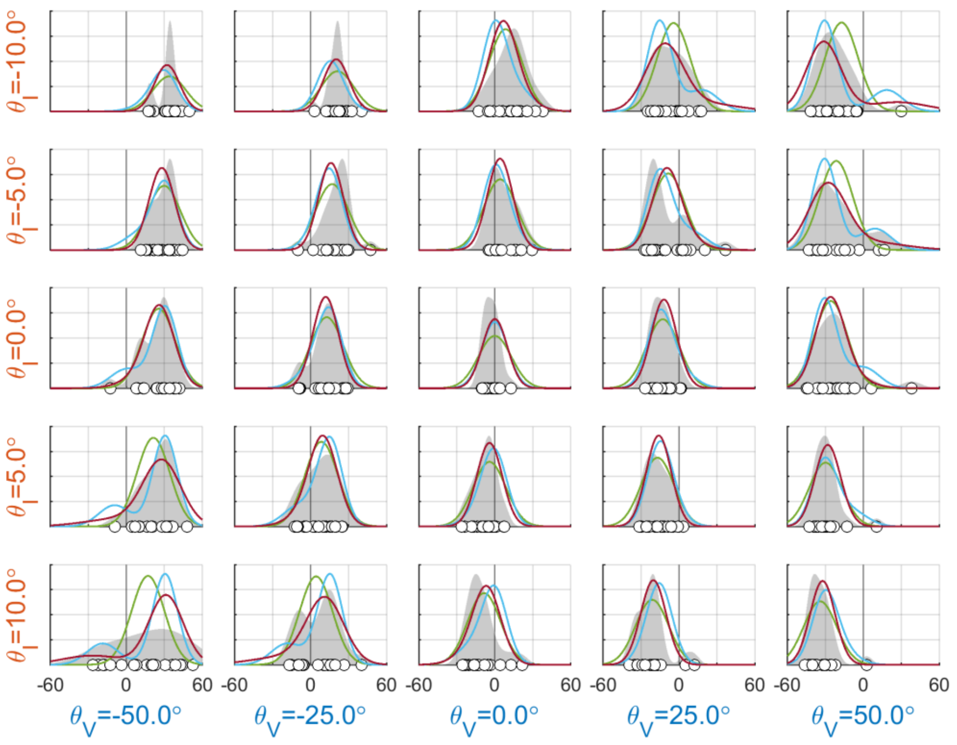

Supplementary material Figure S31. Data and models fits for participant 31 (It. 3)

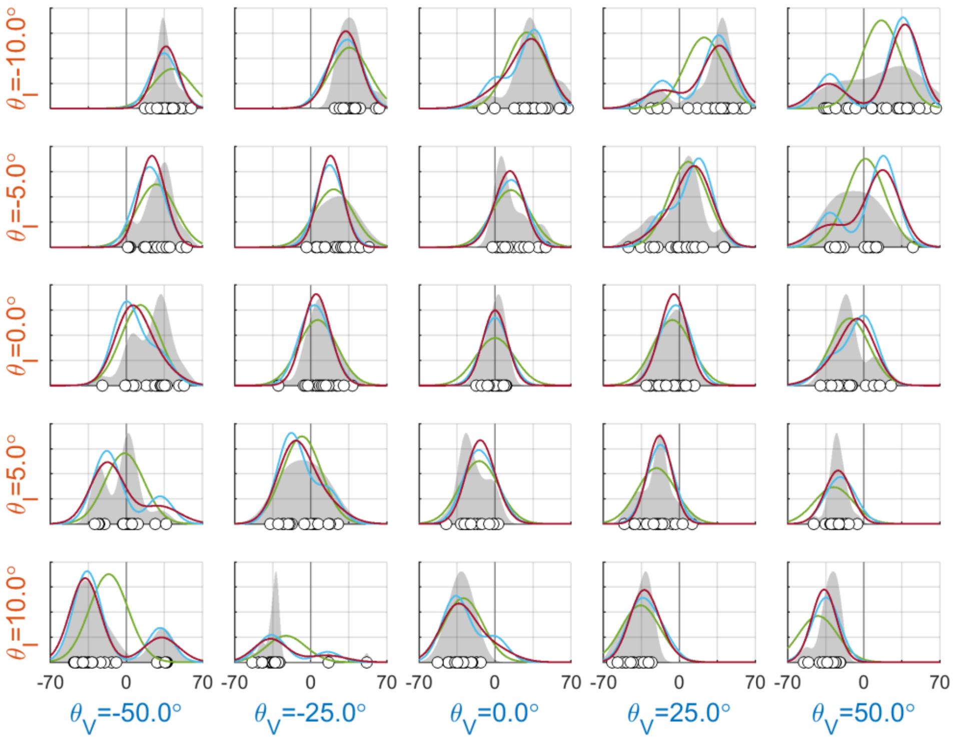

Supplementary material Figure S32. Data and models fits for participant 32 (It. 3)

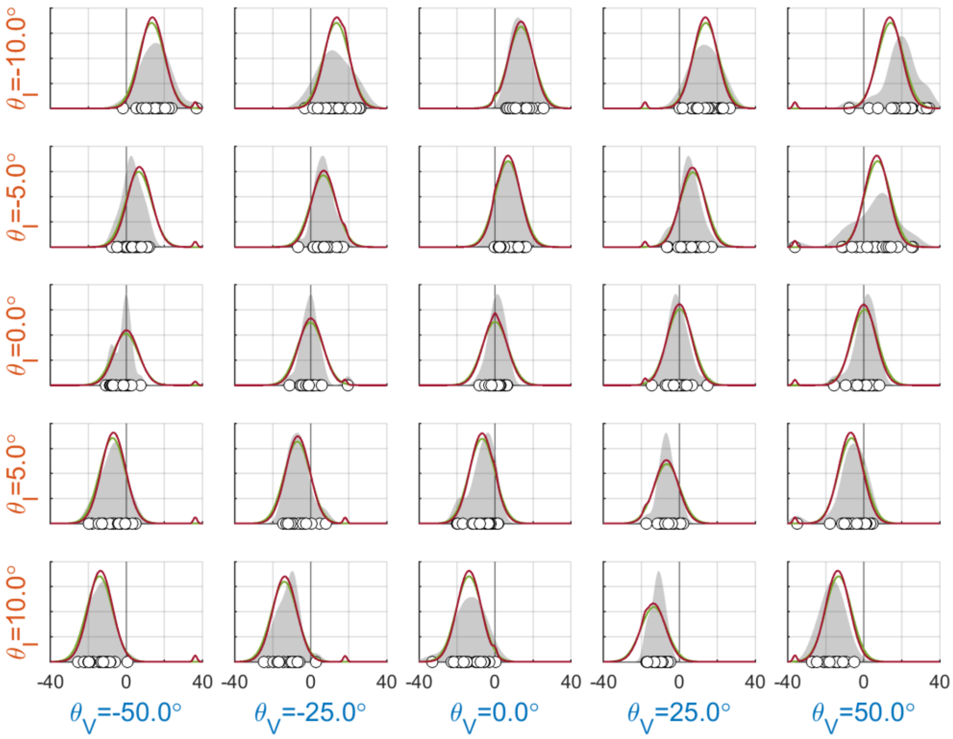

Supplementary material Figure S33. Data and models fits for participant 33 (It. 3)

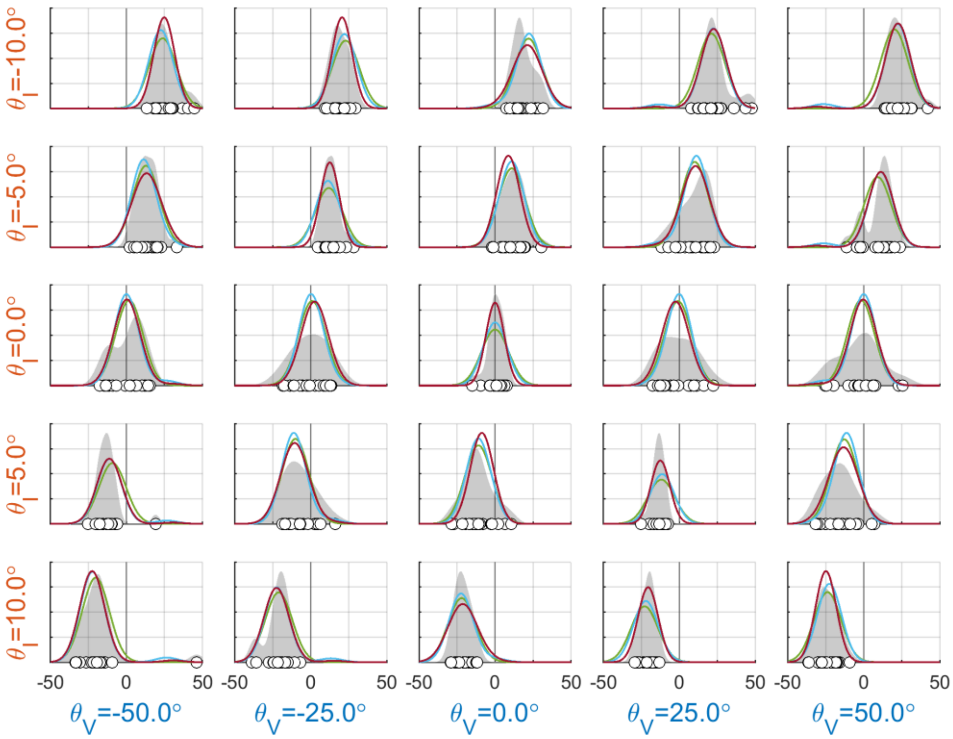

Supplementary material Figure S34. Data and models fits for participant 34 (It. 3)

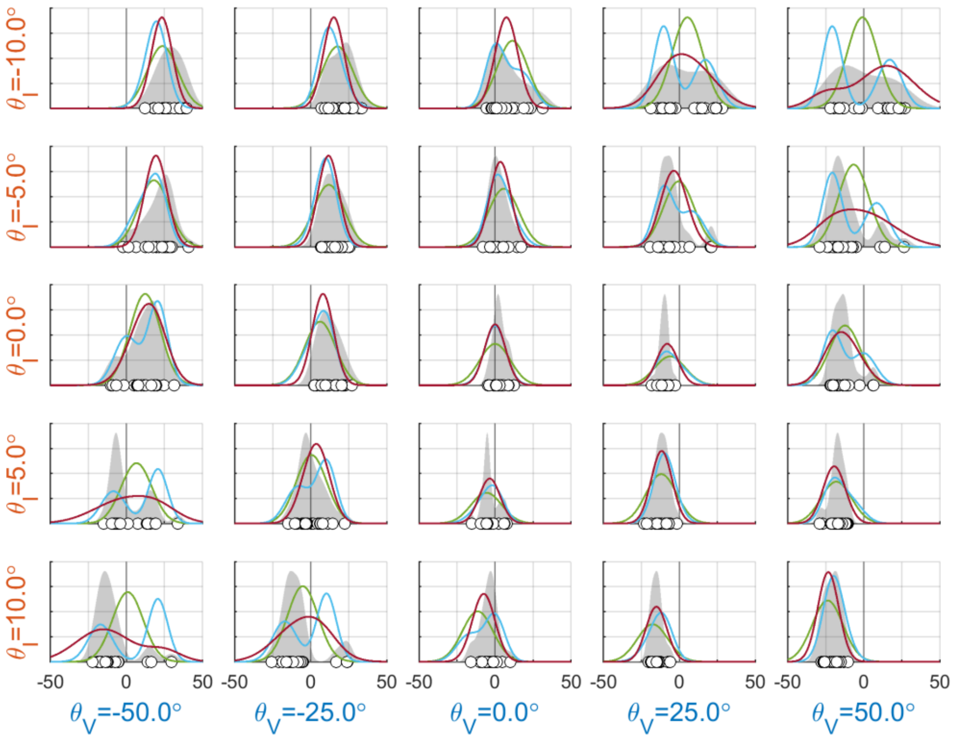

Supplementary material Figure S35. Data and models fits for participant 35 (It. 3)

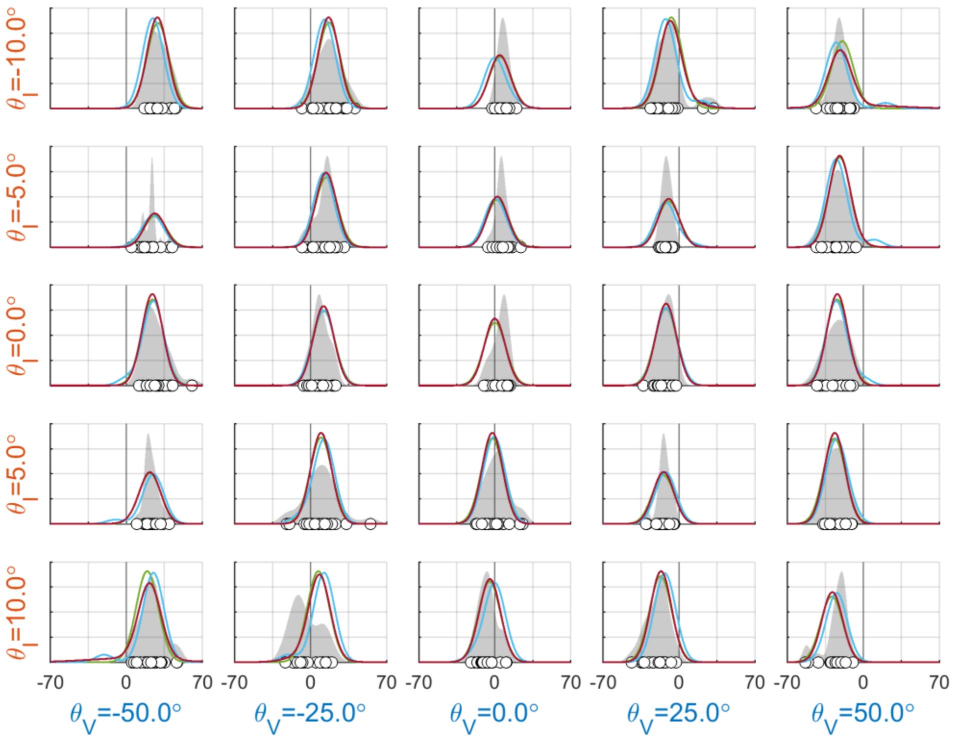

Supplementary material Figure S36. Data and models fits for participant 36 (It. 3)

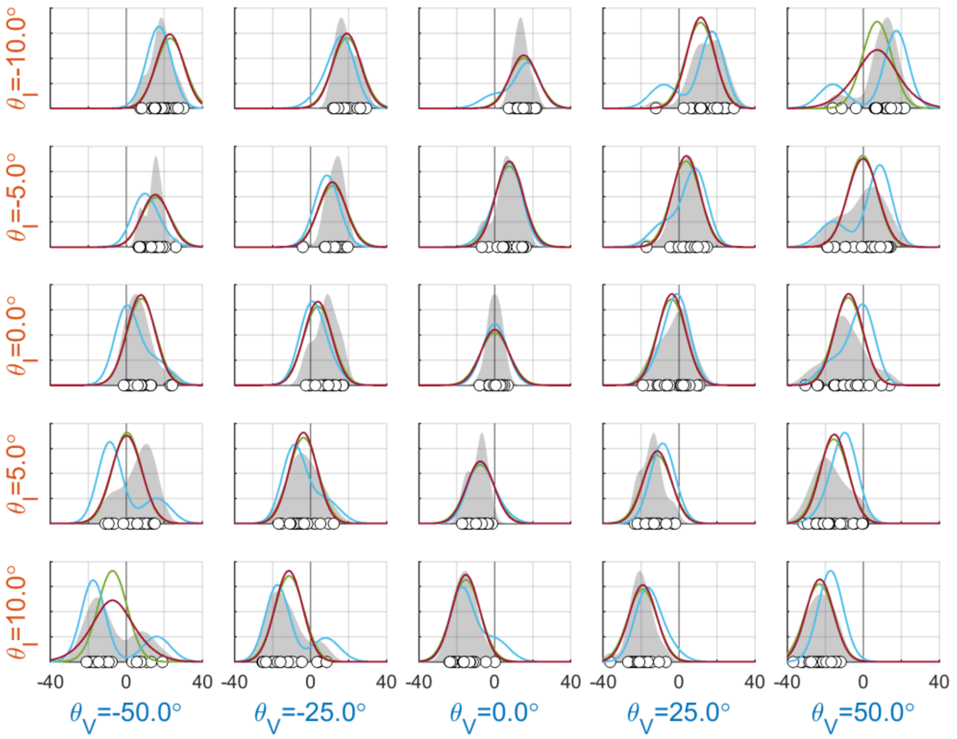

Supplement: Supplementary file 1 — Supplementary Information [file 41598_2018_23838_MOESM1_ESM.pdf]
